# Photoinduced asymmetric charge trapping in a symmetric tetraazapyrene-fused bis(tetrathiafulvalene) conjugate†

**DOI:** 10.1039/d3sc03184e

**Published:** 2023-10-25

**Authors:** Ping Zhou, Maryam Nazari Haghighi Pashaki, Hans-Martin Frey, Andreas Hauser, Silvio Decurtins, Andrea Cannizzo, Thomas Feurer, Robert Häner, Ulrich Aschauer, Shi-Xia Liu

**Affiliations:** a Department of Chemistry, Biochemistry and Pharmaceutical Sciences, University of Bern Freiestrasse 3 CH-3012 Bern Switzerland shi-xia.liu@unibe.ch; b Institute of Applied Physics, University of Bern Sidlerstrasse 5 CH-3012 Bern Switzerland andrea.cannizzo@unibe.ch; c Department of Physical Chemistry, University of Geneva 30 Quai Ernest Ansermet CH-1211 Geneva Switzerland; d Department of Chemistry and Physics of Materials, University of Salzburg Jakob-Haringer-Straße 2A 5020 Salzburg Austria ulrichjohannes.aschauer@plus.ac.at

## Abstract

In fused donor–acceptor (D–A) ensembles, rapid charge recombination often occurs because the D and A units are spatially close and strongly coupled. To the best of our knowledge, a long-lived charge separated (CS) state is still elusive in such systems. The results presented here show that symmetric annulation of two tetrathiafulvalene (TTF) donors to a central tetraazapyrene (TAP) acceptor *via* two quinoxaline units leads to a CS state lifetime of a few ns. A detailed study of the electronic interactions between TTF and TAP units in the ground and excited states was performed and compared with the asymmetric counterpart by cyclic voltammetry, optical absorption and ultrafast transient absorption spectroscopy. The results demonstrate that the photoinduced asymmetric charge trapping between two TTFs significantly stabilizes the CS state, which is also verified theoretically.

## Abstract 2

Asymmetric charge trapping in a symmetric TTF–TAP–TTF leads to a significant stabilization of the charge-separated state of a few ns, which contrasts strongly with the asymmetric TAP–TTF where rapid charge recombination occurs within 2 ps.

## Graphical abstract

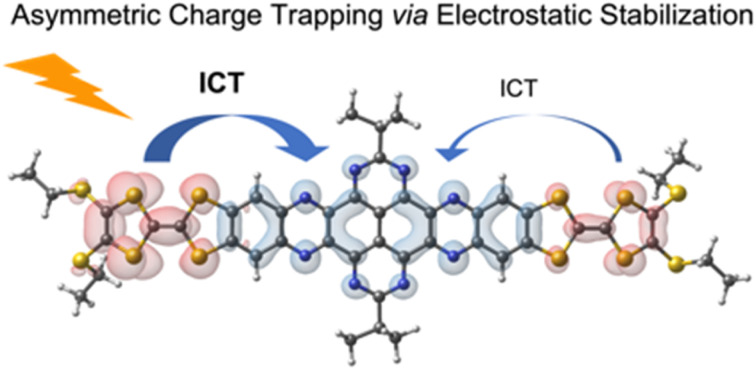

## 1 Introduction

Nitrogenated polycyclic aromatic hydrocarbons (N-PAHs) have attracted much attention due to their unique optoelectronic and electrochemical properties.^1^ Among them, 1,3,6,8-tetraazapyrene (TAP),^2^ a not yet widely used acceptor compound, possesses great potential for the field of organic (opto)electronics as a strong N-rich π-electron acceptor. A promising strategy to develop high-performance organic transistors, photovoltaics, sensors, and switches involves the conjugation of an electron-rich donor (D) and an electron-deficient acceptor (A), which can lead to the formation of an energetically low-lying intramolecular charge-transfer state (ICT) upon photoexcitation.^3,4^ Over the last years, we have focused on a variety of fused D–A ensembles consisting of tetrathiafulvalene (TTF) as the redox-active donor and easily functionalized N-PAH acceptors, such as perylene diimide, hexaazatriphenylene, dipyrido[3,2-*a*:2,3-*c*]phenazine (DPPZ) and others.^5^ The structurally rigid, planar configuration of the resulting D–A systems leads to a high polarizability and an effective extended conjugation, which, in turn, favor intramolecular electronic interactions between TTF and the N-PAH moiety and, hence, pronounced ICT processes. For example, the photoinduced ICT direction can be regulated by either fluoride binding or the oxidation state of a TTF-fused dipyrrolylquinoxaline difluoroborate dyad.^6^ In a different report, the ultrafast optical control of an ICT from TTF to DPPZ units and a metal-to-ligand CT in a *fac*-[Re(CO)_3_(TTF–DPPZ)Cl] complex^7^ describes an elegant approach to an optoelectronic molecular switch triggered by light of different wavelengths. Moreover, TTF-fused D–A systems show a broader range of applications. Among others, they can be used as active materials for positive electrodes in lithium-ion batteries (LIBs),^8^ as efficient CT antennas to sensitize Yb^3+^ and Er^3+^ NIR luminescence,^9^ as semiconducting materials in photovoltaics,^10^ and as organic field-effect transistors.^11^

**Scheme 1:**
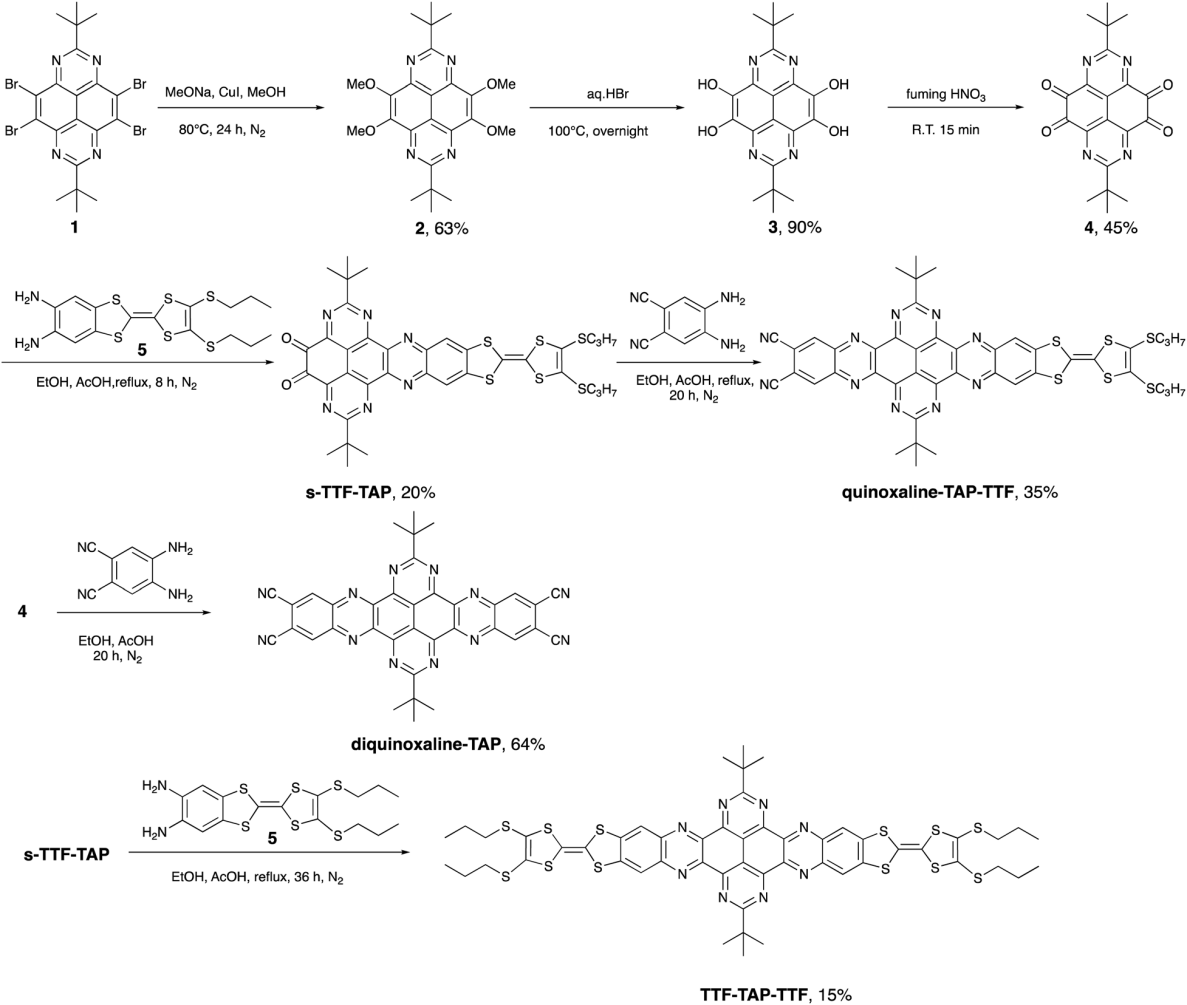
Synthetic routes to asymmetrically and symmetrically functionalized TAP derivatives.

TAP on the other hand, is much less known as a building block of D–A conjugates. This is primarily due to synthetic challenges in the preparation and derivatization of the TAP scaffold. Recently, we reported the synthesis of 2,7-di-*tert*-butyl-1,3,6,8-tetraazapyrene (1, Scheme 1), which serves as a key intermediate in the symmetrically functionalized of TAP with two TTF units yielding a non-fused triad.^12^ No effective electronic communication was observed between these two redox units, as corroborated by the presence of two weak broad absorption bands caused by ICT transitions from HOMOs localized on the peripheral TTF units to the LUMO localized on the central TAP core. To enhance the intramolecular electronic interactions between D and A units, we have embarked on the design and synthesis of linear TAP-annulated mono- and bis(TTF) conjugates. In this work, we report the conversion of 1 to 2,7-di-*t*-butyl-4,5,9,10-tetraone-1,3,6,8-tetraazapyrene (4), which is further elaborated *via* a Schiff-base reaction using 5,6-diamino-2-(4,5-bis(propylthio)-1,3-dithio-2-ylidene)benzo[*d*]-1,3-dithiole (5) or 4,5-diaminophthalonitrile to the D–A systems shown in Scheme 1 (quinoxaline–TAP–TTF, TTF–TAP–TTF and diquinoxaline–TAP). The optical and electrochemical properties of these novel systems were studied in detail. A significant increase in the intensity of an ICT transition was observed in quinoxaline–TAP–TTF and TTF–TAP–TTF. Transient absorption studies revealed the occurrence of ultrafast charge separation and recombination in quinoxaline–TAP–TTF, presumably due to the close proximity between the TTF and TAP entities. In contrast, TTF–TAP–TTF shows a long-lived charge-separated (CS) state, more than two orders of magnitude higher than that of quinoxaline–TAP–TTF, which is attributed to electrostatic stabilization by electronic interactions of the two TTF units. These findings may lead to new approaches for stabilizing the CS states in symmetric quadrupolar D–A–D conjugates.

## 2 Results and discussion

### 2.1 Synthesis and characterisation

As illustrated in Scheme 1, our synthetic approach for the target compounds starts with a nucleophilic aromatic substitution of 2,7-di-*t*-butyl-4,5,9,10-tetrabromo-1,3,6,8-tetraazapyrene (1) with methoxy anions, leading to 2,7-di-*t*-butyl-4,5,9,10-tetramethoxy-1,3,6,8-tetraazapyrene (2) in 63% yield. The key intermediate, 2,7-di-*t*-butyl-4,5,9,10-tetraone-1,3,6,8-tetraazapyrene (4), was obtained in 45% yield after acidic hydrolysis of 2 in hydrobromic acid and subsequent oxidation of the obtained 2,7-di-*t*-butyl-4,5,9,10-tetrahydroxy-1,3,6,8-tetraazapyrene (3) by treatment with fuming nitric acid. The desired target compounds, quinoxaline–TAP–TTF, diquinoxaline–TAP and TTF–TAP–TTF, were prepared in acceptable yields *via* direct condensation of the TTF precursor 5 and/or 4,5-diaminophthalonitrile with precursor 4 in the presence of acetic acid/EtOH. All new TAP building blocks and products were obtained in pure form by simple filtration or chromatographic purification. All products were unambiguously characterized by ^1^H and ^13^C NMR, cyclic voltammetry, UV-Vis-NIR spectroscopy, and high-resolution mass spectrometry. All analytical data match well with their chemical structures.

### 2.2 Electrochemical properties

The electrochemical properties of quinoxaline–TAP–TTF, TTF–TAP–TTF and the reference compounds diquinoxaline–TAP, s-TTF–TAP, 5 and the non-brominated 1 were investigated by cyclic voltammetry in CH_2_Cl_2_ (Fig. S1–S3†). As summarized in Table 1, quinoxaline–TAP–TTF undergoes two distinct reversible oxidations at 0.86 V and 1.22 V and reductions at −0.37 V and −0.92 V. The former corresponds to the formation of TTF radical cation and dication species, while the latter is attributed to the consecutive addition of electrons to the quinoxaline-fused TAP core. Similarly, the symmetric TTF–TAP–TTF undergoes two reversible oxidation processes at 0.89 V and 1.24 V, suggesting that the two TTF units behave as independent redox centres and are simultaneously oxidized to their radical cation and dication species, respectively. Remarkably, diquinoxaline–TAP shows a significant shift to a less negative potential (anodic) compared to non-brominated 1, leading to the occurrence of the second reduction process in an accessible window. This points to the large π-extended conjugation of the TAP-fused bis(quinoxaline-6,7-dicarbonitrile). The same is true for the largest π-conjugate TTF–TAP–TTF among the studied molecules, for which three reversible reductions were observed at −0.42 V, −0.79 V, and −1.02 V, respectively. Apparently, the diquinoxaline-fused TAP core renders quinoxaline–TAP–TTF, TTF–TAP–TTF and diquinoxaline–TAP such strong π-electron acceptors.

**Table 1:** Electrochemical data of the target compounds and precursors. Redox potentials (V) *vs.* Ag/AgCl in CH_2_Cl_2_

Compounds	*E* ^ox1^_1/2_ (V)	*E* ^ox2^_1/2_ (V)	*E* ^red1^_1/2_ (V)	*E* ^red2^_1/2_ (V)	*E* ^red3^_1/2_ (V)	HOMO^a^ (eV)	LUMO^a^ (eV)	*E*_g_ b (eV)
Quinoxaline–TAP–TTF	0.86	1.22	−0.37	−0.92		−5.07	−3.96	1.11
TTF–TAP–TTF	0.89	1.24	−0.42	−0.79	−1.02	−5.06	−3.90	1.16
Diquinoxaline–TAP			−0.31	−0.63			−4.03	
s-TTF–TAP	0.83	1.19	−1.11			−5.03	−3.27	1.76
5	0.37	0.75				−4.62		
TTF	0.41^4^	0.71^4^						
Non-brominated 1			−1.05				−3.31	

a *E*_HOMO_ = −e (*E*_ox_ + 4.27), *E*_LUMO_ = −e (*E*_red_ + 4.27), *E*_ox_ = the onset electrochemical potential of oxidation, *E*_red_ = the onset reduction potential, Fc/Fc^+^ is 0.53 V relative to Ag/AgCl in CH_2_Cl_2_.

b *E*_g_ = *E*_LUMO_ − *E*_HOMO_.

The fusion of D and A units often leads to the stabilization of the HOMO and the destabilization of the LUMO, which is confirmed by a positive shift of the first electrochemical potential of oxidation and a negative shift of the first reduction potentials with respect to the redox potentials of the D and A components. This statement holds true only if the locations of HOMO and LUMO remain almost unchanged by large π-extended conjugation. Compared to the reference compounds TTF and 5, the first electrochemical potentials of oxidation of s-TTF–TAP, quinoxaline–TAP–TTF and TTF–TAP–TTF are positively shifted by more than 400 mV due to the electron-withdrawing effect of the quinoxaline-fused TAP core. With reference to diquinoxaline–TAP, the replacement of CN groups by electron-donating TTF ring(s) leads to a noticeable cathodic shift of the first reduction potential. The same electron-donating effect of TTF is also demonstrated by direct comparison of s-TTF–TAP to non-brominated 1 where the LUMO is mainly localized on the TAP core.

Both HOMO and LUMO as well as electrochemical HOMO–LUMO gaps are estimated (Table 1), indicating that the addition of the second TTF has a negligible effect on the HOMO.

### 2.3 Optical properties

**Fig. 1:**
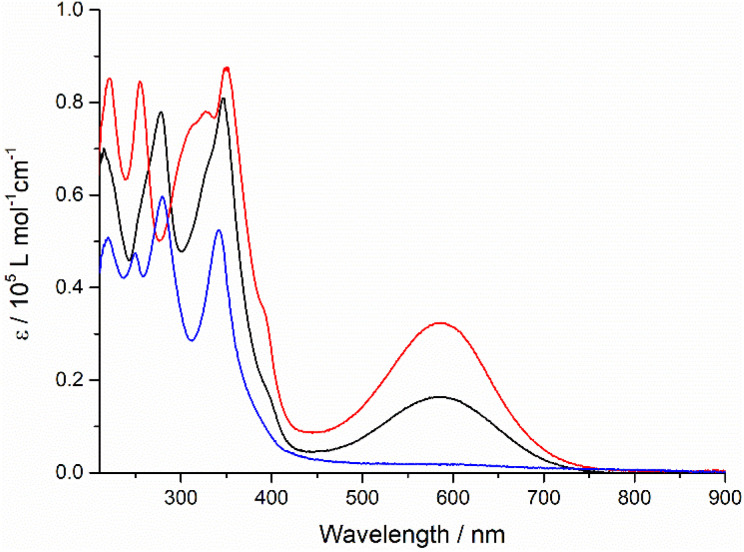
The UV-Vis absorption spectra of quinoxaline–TAP–TTF (black), TTF–TAP–TTF (red) and diquinoxaline–TAP (blue) (*c* = 5 × 10^−6^ M) in THF at r.t.

A strong and broad absorption band peaking at 587 nm is attributed to an ICT transition from the TTF unit to the quinoxaline-fused TAP moiety, since it is not given only by the sum of the optical spectra of the individual TAP and TTF components.^12,13^ The extinction coefficient of this ICT band increases from 1.6 × 10^4^ to 3.2 × 10^4^ M^−1^ cm^−1^ on going from one to two TTF units per conjugate. In the UV and blue parts of the optical spectra, a series of very intense absorption bands is observed in a range of 230–400 nm. Compared to diquinoxaline–TAP (blue curve, Fig. 1), they are attributed to several overlapping π–π* transitions localized on the TTF and TAP cores, as well as ICT transitions. It is worth noting that TTF–TAP–TTF (red curve, Fig. 1) has an additional strong band around 320 nm in contrast to quinoxaline–TAP–TTF (black curve, Fig. 1). All these spectral features indicate that the annulation of one more TTF to the central quinoxaline-fused TAP core significantly extends the π-conjugation, not only leading to an electron redistribution between two TTF units but also affecting the oscillator strengths of the different transitions to a large extent. These experimental results are further verified by DFT calculations (see below).

**Fig. 2:**
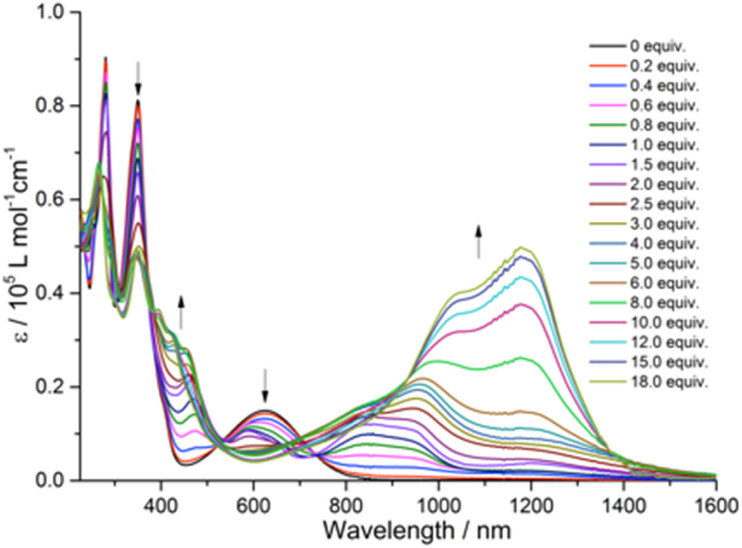
Variation of UV-Vis-NIR absorption spectra of quinoxaline–TAP–TTF (5 × 10^−6^ M) in CH_2_Cl_2_ upon successive addition of aliquots of NOSbF_6_ at r.t.

To understand the intramolecular electronic interactions between the TTF and quinoxaline-fused TAP in the quinoxaline–TAP–TTF and TTF–TAP–TTF, the UV-Vis-NIR spectroscopy was applied to monitor their spectral variations upon chemical oxidation using aliquots of NOSbF_6_ (Fig. 2 and S4†). As NOSbF_6_ has a redox potential of +0.87 V (*vs.* Fc^+^/Fc), it can act as an oxidizing reagent for the oxidation of TTF units.^14^ Upon successive addition of NOSbF_6_, quinoxaline–TAP–TTF is oxidized to form the corresponding radical species quinoxaline–TAP–TTF˙^+^. As a result, a progressive decrease in the absorbance of both π–π* and ICT transitions at 320 nm and 620 nm, respectively, is accompanied with a concomitant emergence of absorption bands at 450 nm and 900 nm, which reach their maximum values upon addition of 1.4 equiv. of NOSbF_6_ (Fig. 2). These two new absorption bands are characteristic of the TTF˙^+^ radical cation-related transitions within a D–A ensemble.^15^ Moreover, as the amount of NOSbF_6_ increases, a strong broad absorption band gradually evolves in the 800–1400 nm range, which remains unchanged upon addition of 18 equiv. of NOSbF_6_. This band can be attributed to the dimeric radical cation species arising from enforced intermolecular interactions.^16^

Likewise, the initial oxidation of TTF–TAP–TTF (Fig. S4†) results in the appearance of two absorption bands at 450 nm and 900 nm, indicating the formation of the diradical species ˙^+^TTF–TAP–TTF˙^+^ due to the spontaneous oxidation of two TTF units, as confirmed by the CV measurements. In contrast to quinoxaline–TAP–TTF, an additional, very weak, broad absorption band peaking at 1400 nm is synchronously generated, tentatively ascribed to a mixed-valence charge transfer transition from neutral TTF–TAP–TTF to ˙^+^TTF–TAP–TTF˙^+^ diradical species.^14,17^ This lowest energy absorption band is continuously blue-shifted during the further oxidation process and reaches its maximum value around 1200 nm upon addition of 18 equiv. of NOSbF_6_. This observation suggests that spontaneous self-association of the ˙^+^TTF–TAP–TTF˙^+^ diradical to its dimeric species occurs, triggered by a strong π–π interaction between large planar π-conjugates. It can be deduced, therefore, that both quinoxaline–TAP–TTF and TTF–TAP–TTF exhibit a strong propensity for π-dimerization, as the attractive π–π interaction over long distances outweighs the electrostatic repulsion.^18^

### 2.4 Computational study

**Fig. 3:**
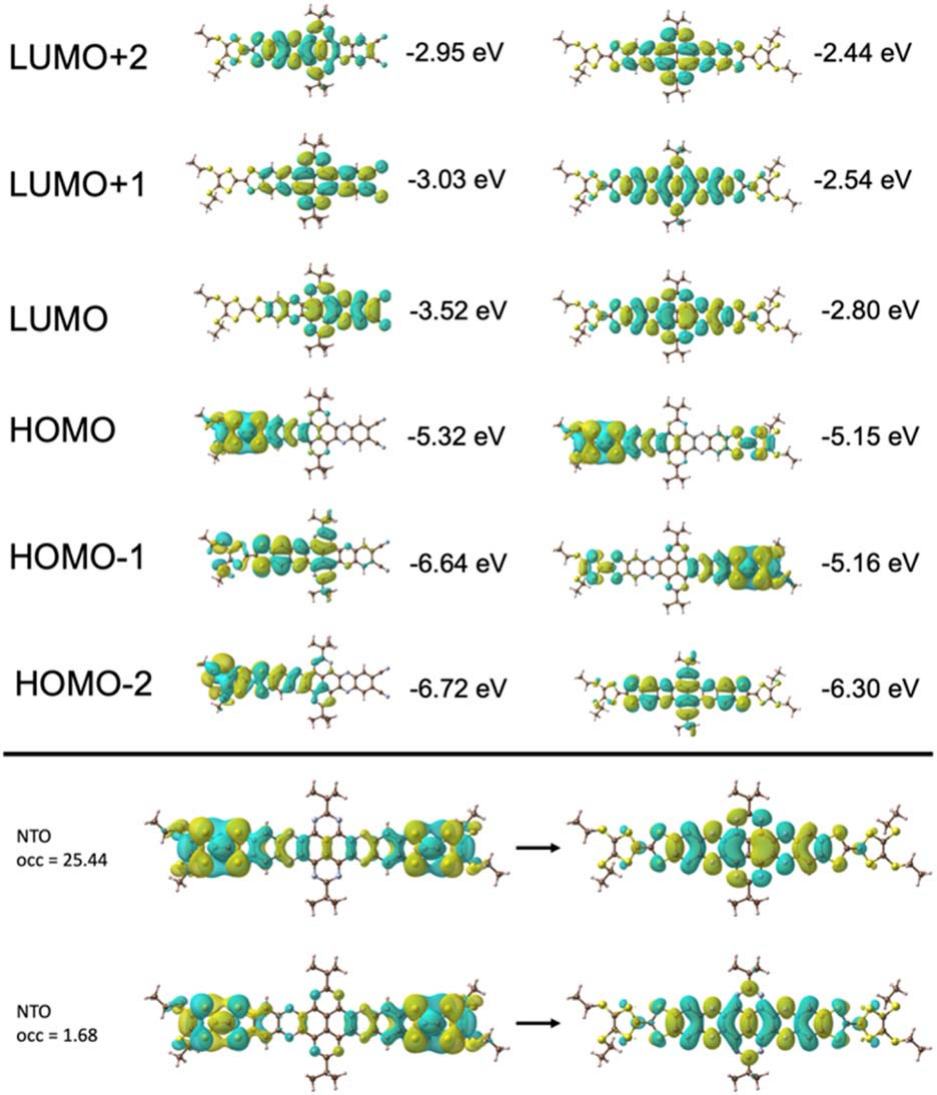
Molecular orbitals of quinoxaline–TAP–TTF (top-left) and TTF–TAP–TTF (top-right) that are involved in the ICT transitions in the visible spectral region and MO energies are also given. Natural transition orbitals (NTO) of TTF–TAP–TTF (bottom) for the two combinations with the highest contribution (characterized by their occupation (occ) parameter) for the S_0_ → S_1_ transition.

TD-DFT calculations of quinoxaline–TAP–TTF and TTF–TAP–TTF were performed at the B3LYP/6-31G(d,p) level of theory to predict various electronic transitions (Tables S1 and S2†). In quinoxaline–TAP–TTF, S_0_ → S_1_ and S_0_ → S_2_ transitions in the low energy region (783 nm and 610 nm in vacuum, respectively, Table S1†) reflect an ICT nature. The former is dominated by electron transfer solely from the HOMO localized mainly on the TTF with certain contribution from the adjacent quinoxaline unit to the LUMO on the quinoxaline-fused TAP weighted towards the quinoxaline-6,7-dicarbonitrile part. The latter is almost exclusively (99%) attributed to the excitation from HOMO to LUMO+1 delocalized over the CN-extended quinoxaline-fused TAP core (Fig. 3 and S5†). Both, however, exhibit a very weak oscillator strength of (0.05 or 0.004) and are not expected to be readily observed. A much larger oscillator strength of 0.32 is associated with the S_0_ → S_3_ transition at 604 nm, consisting of a nearly pure HOMO → LUMO+2 (99%) transition. The LUMO+2 spreads broadly over the quinoxaline-fused TAP core, weighted towards the quinoxaline part close to the TTF unit.

As expected, HOMO and HOMO−1 are nearly degenerate in symmetric TTF–TAP–TTF. As depicted in Fig. 3, they are localized on one TTF unit with some contribution from the other TTF unit. The LUMOs are distributed across the whole quinoxaline-fused TAP core with a significant contribution from the four S atoms. The lowest energy absorption band at 608 nm in vacuum is assigned to the S_0_ → S_1_ transition (Table S2†). This electronic excitation is predominated by charge migration from the HOMO → LUMO (79%). In addition, HOMO−1 → LUMO (15%) and HOMO−1 → LUMO+1 (4%) are involved. The molecular orbitals LUMO and LUMO+1 are symmetric with respect to both TTF units, whereas the HOMO−1 and HOMO are not. From the percentages given, a stronger depletion of the left TTF than the right one is predicted in this ICT process. This slight asymmetry is also clearly visible in the natural transition orbitals (Fig. 3, bottom), where the pair with the highest contribution (occ = 25.44), shows a significantly larger contribution of charge moving from the left TTF to a fairly symmetric distribution on the TAP core. Despite this asymmetry, the partial hole localization on both TTF units in the excited state, however, yields a more symmetric electrostatic potential energy landscape compared to an excited state with the hole on a single TTF as it occurs for quinoxaline–TAP–TTF. This more symmetric electrostatic potential energy landscape (Fig. S6†) is expected to electrostatically stabilize the excited electron on the quinoxaline-fused TAP core, which accounts for a long-lived CS state observed in the following transient absorption measurements. In the ground state, the two TTF are however equivalent in terms of the nearly degenerate HOMO and HOMO−1, which agrees well with the electrochemical properties. As aforementioned, the two TTF units become oxidized at the same potential because one electron is likely removed from each of the HOMO and HOMO−1 rather than two from the HOMO. Taking the oscillator strength of 0.62 into account, the S_0_ → S_1_ transition in TTF–TAP–TTF has an amplitude almost twice as large as the S_0_ → S_3_ transition in quinoxaline–TAP–TTF, despite their ICT nature and almost same energy. This perfectly matches the experimental results shown in Fig. 1.

In both compounds, π–π* and ICT transitions with large oscillator strengths are estimated in the high energy spectral region below 400 nm. Overall, their predicted absorption spectra are in quite good agreement with the experimental results (Fig. S7†). It is worth noting that the absorption spectra were computed in vacuum and that in general a slight red shift is observed in solvents, slightly more so in polar solvents as shown in Fig. S8.†

### 2.5 Transient absorption spectroscopy

**Fig. 4:**
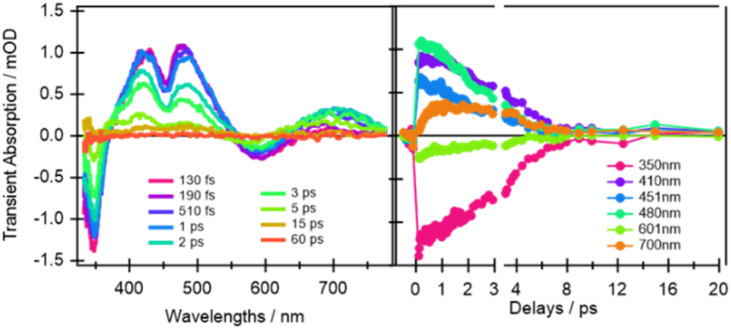
Selection of ultrafast transient absorption spectra and kinetics of quinoxaline–TAP–TTF in THF excited at 580 nm at different time delays (left) and wavelengths (right). The analysis in these date is reported in the ESI.†

From the earliest spectrum (130 fs after excitation), the transient absorption (TA) spectra of quinoxaline–TAP–TTF (Fig. 4, left) show an ICT ground state bleach (GSB) signal at 580 nm, accompanied by a GSB at 350 nm and three excited-state absorption (ESAs) peaks at 400 nm, 480 nm and 650–700 nm. These spectral changes upon excitation of the ICT state are in excellent agreement with the changes induced by chemical oxidation of quinoxaline–TAP–TTF (Fig. 2): the rise of an absorption signal around at 450 nm and at *λ* > 700 nm, with a concomitant reduction of the ICT and 350 nm bands. This allows us to assign the GSB at 350 nm and the ESA bands to the oxidation of the TTF unit which is also corroborated by the ultrafast investigation of the diquinoxaline–TAP upon UV (355 nm) excitation (Fig. S9†). Accordingly, the red-shift from 660 nm to 700 nm of the red-most ESA band can be rationalized as the effect of solvation dynamics driving the stabilization of the ICT state in *ca.* 1 ps.^19^

Concerning dynamics, kinetic traces in Fig. 4 (right) and decay associated spectra (DAS) in Fig. S10† allow us to estimate the lifetime of the excited states, and therefore the time constant of the back charge transfer process, to be *ca.* 2–2.5 ps. A thorough observation of the kinetic traces in Fig. 4 also reveals that the signal decays non-exponentially at any wavelength. This agrees with the occurrence of several processes with opposite effects on the signal strength, such as solvation, cooling, conformational relaxation, stabilization of the charge separation state, on the same timescale as the back charge transfer. This is manifested in the result of the Singular Value Decomposition and Global Fit (SVD-GF) analysis, which shows two dynamics with *τ*_1_ ∼ (1.3 ± 0.2) ps and *τ*_2_ ∼ (2.2 ± 0.2) ps. At first superficial glance, it could be rationalized by assuming a rise of the overall signal due to the formation of the ICT state, followed by a charge recombination in 2–2.5 ps, but this is in contradiction with the direct nature of the ICT transition. Instead, as aforesaid, this is due to the limitation of the SVD-GF analysis in describing non-exponential dynamics or exponential decays with close time constants.

In other words, the 1.3 ps DAS describes more an average effect of several processes which are not by themselves related to a change in the population of the CT state but to a modulation of the total signal strength. Therefore, despite the relative DAS would formally describe a rise of the signal, it should be more considered as describing a deviation from a pure exponential decay. The value of 1.3 has still the physical meaning of an average time scale of the processes competing with the back CT one, in agreement with analysis of the TTF–TAP–TTF (see below).

**Fig. 5:**
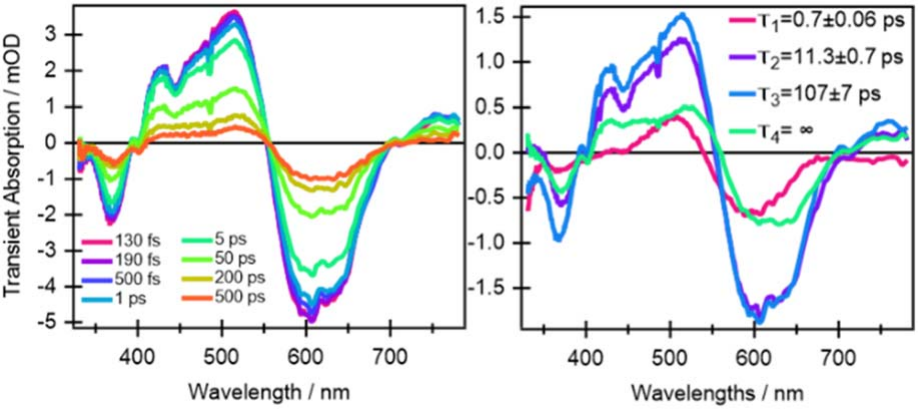
Selection of ultrafast transient absorption spectra of TTF–TAP–TTF excited at 605 nm at different time delays in THF (left) and DAS with relative decay time constants (right, see the main text for the assignments of the spectral bands).

In comparison to quinoxaline–TAP–TTF, TTF–TAP–TTF displays TA spectra that are similar overall, albeit not the same (Fig. 5, left): a red-shift of the GSB at 350 nm to 375 nm, revealing another GSB at *λ* < 350 nm and a broader ESA at *λ* > 450 nm, which is related to the oxidized TTF and in agreement with the experimental data in Fig. S4.† The results of SVD-GF analysis identify four main dynamics (Fig. 5 and S12†): the 700 fs DAS describes a shift and the appearance of a band mainly due to the solvation and stabilization of the ICT state (in agreement with the qualitative discussion of the kinetics in quinoxaline–TAP–TTF). Since we detected at the magic angle, all three components describe population decays, energy redistribution and reorganization of the electron density at 11 ps, 107 ps and infinity (relative to the measurement time window). They have a similar shape with a distinct GSB band at 630 nm. This means that both the 11 ps and 107 ps components mainly describe a back charge transfer of ∼80% of the molecules and the remaining 15% of the population decays on a time scale longer than few ns. These results indicate that the back charge transfer in TTF–TAP–TTF is much longer than in quinoxaline–TAP–TTF. The 11 ps and 107 ps components show a slightly stronger ESA signal at 525 nm than the long-lived component, which would point to a long-lived conformational or electronic state distinct from the intermediate ones. On the other hand, the strong similarity of the 11 ps and 107 ps DASs would speak for the same electronic state, likely with a slightly different extent of distortion.

In principle, the amplitude of the ESA signals at 480 nm (Fig. 4) and 530 nm (Fig. 5) can be used as an estimation of the oxidized TTF signal for quinoxaline–TAP–TTF and TTF–TAP–TTF, respectively, since this region contains the smallest GSB and the lowest possible contribution from the TAP core according to their optical absorption spectra (Fig. 1). Similarly, the amplitude of the signal at the maximum of the ICT band (580 nm and 630 nm, for quinoxaline–TAP–TTF and TTF–TAP–TTF, respectively) can be used to monitor the amount of ICT GSB. Normalizing the signals of the ICT GSB to their corresponding oxidized TTF (5 mOD/2.75 mOD = 1.85 and 0.5 mOD/0.75 mOD = 0.66, in TTF–TAP–TTF and quinoxaline–TAP–TTF, respectively), indicates that the GSB of the ICT transition in TTF–TAP–TTF is more than twice that in quinoxaline–TAP–TTF. This observation can be rationalized by the fact that one TTF makes a much larger contribution to the ICT transition than the other. On the other hand, the insertion of one more TTF leads to a significant change in the oscillator strength of the ICT transition, as corroborated by above-mentioned DFT calculations.

**Fig. 6:**
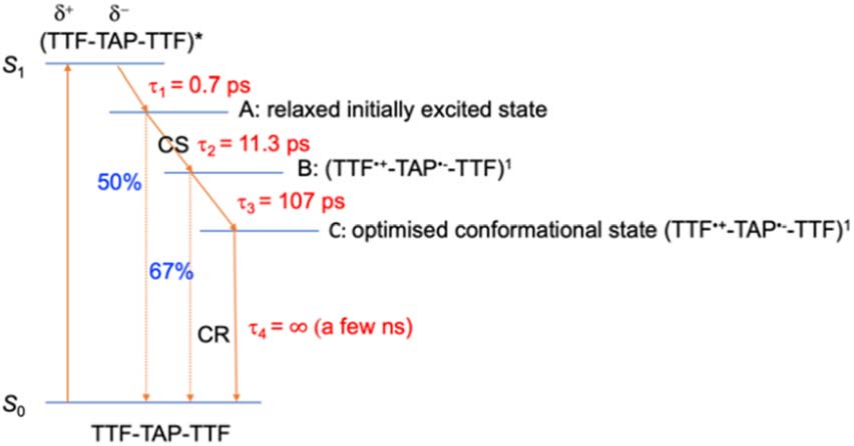
Schematic representation of the relaxation processes for TTF–TAP–TTF upon excitation at 580 nm into the ICT band. (A) Relaxed initially excited state, (B) singlet charge-separated state, (C) optimised conformational singlet charge-separated state.

Another point is that the oxidized signal of TTF in quinoxaline–TAP–TTF is *ca.* 4 times lower compared to TTF–TAP–TTF. This can be estimated by comparing the ratio of ESA at 480 nm in quinoxaline–TAP–TTF and at 530 nm in TTF–TAP–TTF at 130 fs, which results in 4 times higher excitation in TTF–TAP–TTF. This means that at least more than 75% of the excited quinoxaline–TAP–TTF relaxes to the ground state before the first collected TA spectrum at 130 fs. This reveals that the ICT recombination in the quinoxaline–TAP–TTF is also at least biexponential, with sub-100 fs and 2 ps components. It can be deduced, therefore, that both compounds show the same biexponential behavior with the quinoxaline–TAP–TTF being 50 times faster. Such observation is verified by the calculation of the plane-averaged electrostatic potential projected along the long molecular axis for the structurally relaxed S_1_ state. As shown in Fig. S6,† the charge recombination for quinoxaline–TAP–TTF is already favorable in the non-relaxed initial S_1_ state (purple curve), which is consistent with its very short lifetime. In contrast, the charge recombination for TTF–TAP–TTF is not favorable in the initial S_1_ state (blue curve) and only becomes so after structural relaxation (red curve), pointing to a long-lived CS state. As mentioned above, this can be explained by electrostatic attraction towards both TTF units in TTF–TAP–TTF that gets reduced during structural relaxation accompanied by hole localization on the left TTF unit. Consequently, the CS state is populated only in TTF–TAP–TTF where the second TTF unit is available, followed by a conformational relaxation leading to a long-lived CS state (Fig. 6).

## 3 Conclusions

An efficient synthetic approach to derivatization of the TAP skeleton is reported, which allows preparing a series of large π-extended conjugates, such as diquinoxaline–TAP, quinoxaline–TAP–TTF, and TTF–TAP–TTF. In conjunction with electrochemical and absorption measurements, it was found that the symmetric TTF–TAP–TTF has a long-lived CS state, in stark contrast to the asymmetric quinoxaline–TAP–TTF. This finding provides a truly ingenious way for prolonging the lifetime of a potential intramolecular charge separation by chemical design. In due course, different kinds of TAP-based D–A systems for a precise control of the light-induced charge flow are going to be investigated in detail, which is of paramount importance for the development of novel materials for energy conversion and storage.

## Data availability

Data for all the new compounds in this manuscript are available in the ESI,† which includes experimental details and characterisation, DFT calculations and additional figures for transient absorption spectra.

## Author contributions

P. Z. synthesized and characterised molecules and with the aid of H.-M. F., M. N. H. P. performed transient absorption spectroscopy measurements and analyzed the data. S.-X. L., S. D., R. H., A. C. and T. F. conceived and co-supervised the project. U. A. carried out theoretical calculations and analyzed the data. P. Z. and M. N. H. P. wrote the initial draft with input from all authors. S.-X. L., A. C. and U. A. prepared the manuscript with the aid of A. H., S. D., R. H. and T. F. All authors have given approval to the final version of the manuscript.

## Conflicts of interest

There are no conflicts to declare.

## Supplementary Material

SC-014-D3SC03184E-s001
