# Supplementary material for: Photoinduced asymmetric charge trapping in a symmetric tetraazapyrene-fused bis(tetrathiafulvalene) conjugate
Source: Chem Sci. 2023 Oct 25;14(44):12715–22. doi: 10.1039/d3sc03184e (PMC10646961; doi:10.1039/d3sc03184e)
Supplement: SC-014-D3SC03184E-s001 [file SC-014-D3SC03184E-s001.pdf]

This journal is © The Royal Society of Chemistry 2023

Experimental Section

Air- and/or water-sensitive reactions were conducted under nitrogen and dry, freshly distilled solvents were used. 2,7-di-*t*-butyl-4,5,9,10-tetrabromo-1,3,6,8-tetraazapyrene (**1**)[1] and 5,6-diamino-2-(4,5-bis(propylthio)-1,3-dithio-2-ylidene)benzo[*d*]-1,3-dithiole (**5**)[2] were prepared as described in the literature. Chemicals used for the synthesis of the compounds were purchased from commercial suppliers (Sigma-Aldrich, TCI or Alfa Aesar). UV-Vis-NIR absorption spectra were recorded on a Perkin Elmer Lambda 900 UV/Vis/NIR spectrometer and UV-Vis absorption spectra on a Varian Cary-100 Bio-UV/VIS. ^{1}H and ^{13}C NMR spectra were recorded on a Bruker Avance 300 or 400 spectrometer at 300 MHz and 75 MHz or 101 MHz, respectively. Chemical shifts are reported in parts per million (ppm) and are referenced to the residual solvent peak ($CDCl_3$, δ ^{1}H = 7.26 ppm, δ ^{13}C = 77.16 ppm, and DMSO-d_6, δ ^{1}H = 2.50 ppm, δ ^{13}C = 39.52 ppm). The following abbreviations were used s (singlet), d (doublet), t (triplet) and m (multiplet). FT-IR data were collected on a FT/IR-4700 FTIR Spectrometer from JASCO. High resolution mass spectra (HR-MS) were obtained on a Thermo Fisher LTQ Orbitrap XL using Nano Electrospray Ionization. Cyclic voltammetry (CV) was performed in a three-electrode cell equipped with a Pt working electrode, a glassy carbon counter-electrode, and Ag/AgCl reference electrode. The electrochemical experiments were carried out under an oxygen-free atmosphere in dichloromethane with $TBAPF_6$ (0.1 M) as a supporting electrolyte.

2,7-di-*t*-butyl-4,5,9,10-tetramethoxy-1,3,6,8-tetraazapyrene (2). 2,7-di-*t*-butyl-4,5,9,10-tetrabromo-1,3,6,8-tetraazapyrene (634 mg, 1 mmol), copper iodide (952 mg, 5 mmol), sodium methoxide solution (702 mg, 13 mmol), and methanol (50 mL) were put into a round-bottom flask (100 ml), and the resultant solution was stirred at 80 °C for 2 days under N_2. After cooling down to room temperature, the reaction was quenched by water (50 ml). The mixture was extracted by dichloromethane (50 mL) for three times. The combined organic phase was washed with an aqueous solution of ammonium chloride, and then the solvent was removed by a rotavapor. The crude product was purified by flash column chromatography (silica gel, CH_2Cl_2) to afford **2** as a yellow solid (278 mg). Yield: 63%. ^{1}H NMR (300 MHz, $CDCl_3$) δ 4.48 (s, 12H), 1.67 (s, 18H). ^{13}C NMR (75 MHz, $CDCl_3$) δ 175.08, 150.31, 149.73, 107.32, 63.08, 40.92, 30.40. IR data (cm^{-1}): 2933, 2902, 2866, 2359, 1548, 1468, 1380, 1206, 990, 801, 471. HR-MS (ESI, positive): *m/z* calcd for $[C_{24}H_{30}N_4O_4+H]^+$: 439.2340; found: 439.2324.

2,7-di-*t*-butyl-4,5,9,10-tetrahydroxy-1,3,6,8-tetraazapyrene (3). A mixture of **2** (79 mg, 0.18 mmol) and 48% hydrobromic acid (10 mL) were put into a round-bottom flask (50 mL), and the reaction solution was stirred at 100 °C overnight under N_2. After the temperature of the reaction mixture was cooled to room temperature, water (20 mL) was added, leading to some precipitation. After filtration, the collected solid was washed with water and dried *in vacuum*. The analytically pure product **3** was obtained as a dark-brown solid (61 mg). Yield: 90%. ^{1}H NMR (300 MHz, $CDCl_3$) δ 1.69 (s, 18H).

^{1}H NMR (300 MHz, DMSO-d_6) δ 10.08 (s, 4H), 1.63 (s, 18H). ^{13}C NMR (101 MHz, DMSO) δ 173.33, 145.93, 140.87, 101.41, 30.26. IR data (cm^{-1}): 3363, 3188, 2956, 2921, 2857, 2361, 1736, 1477, 1302, 983, 836, 686, 497. HR-MS (ESI, positive): *m/z* calcd for $[C_{20}H_{22}N_4O_4+H]^+$: 383.1714; found: 383.1708.

2,7-di-*t*-butyl-4,5,9,10-tetraone-1,3,6,8-tetraazapyrene (4). A mixture of **3** (57 mg, 0.15 mmol) and fuming nitric acid (10 mL) was put into a round-bottom flask (50 mL), and the reaction solution was stirred at room temperature for 15 minutes. Water (16 mL) was added, and then the resultant solution was concentrated. A yellow precipitate was formed and separated by filtration. The obtained yellow solid was redissolved in chlorobenzene (25 mL) and the resulting solution was stirred overnight under reflux. By cooling down to room temperature, the precipitate was formed, filtrated off and then dried *in vacuum*. The analytically pure product **4** was obtained as an orange solid (26 mg). Yield: 45%. ^{1}H NMR (300 MHz, DMSO-d_6) δ 1.47 (s, 18H). ^{13}C NMR (101 MHz, DMSO-d_6) δ 177.41, 174.95, 155.03, 121.11, 29.39. IR data (cm^{-1}): 2965, 2927, 2904, 2361, 2333, 1734, 1704, 1566, 1504, 1478, 1456, 1399, 1357, 1215, 1103, 842, 429. HR-MS (ESI, positive): *m/z* calcd for $[C_{20}H_{18}N_4O_4 + 2H_2O+H]^+$ 415.1612, found 415.1604.

Diquinoxaline-TAP. A mixture of 4,5-diaminophthalonitrile (95 mg, 0.6 mmol) and **4** (76 mg, 0.2 mmol) in EtOH (10 mL) was refluxed for 20 h in the presence of a catalytical amount of acetic acid (2 mL). The resulting precipitate was filtered off, washed with EtOH and diethyl ether and dried *in vacuum* to afford the product as a yellow solid (80 mg). Yield: 64%. ^{1}H NMR (300 MHz, DMSO-d_6) δ 9.59 (s, 4H), 1.81 (s, 18H). HR-MS (ESI, positive): *m/z* calcd for $[C_{36}H_{22}N_{12}+H]^+$: 623.2163; found: 623.2171.

s-TTF-TAP. A mixture of **5** (195 mg, 0.45 mmol) and **4** (189 mg, 0.5 mmol) in EtOH (50 mL) was refluxed for 8 h in the presence of a catalytical amount of acetic acid (5 mL). The resulting precipitate was filtered off, washed with EtOH and diethyl ether and dried *in vacuum*. The crude product was purified by flash column chromatography (silica gel, CH_2Cl_2:EtOAc,20/1, v/v) to afford **s-TTF-TAP** as a blue solid (68 mg). Yield: 19.5%. ^{1}H NMR (300 MHz, $CDCl_3$) δ 8.33 (s, 2H), 2.84 (m, 4H), 1.71-1.68 (m, 22H),, 1.06 (m, 6H). HR-MS (ESI, positive): *m/z* calcd for $C_{36}H_{34}N_6O_2S_6$: 774.1062; found: 774.1078.

TTF-TAP-TTF. A mixture of **5** (80 mg, 0.19 mmol) and **s-TTF-TAP** (72 mg, 0.09 mmol) in EtOH (25 mL) was refluxed for 36 h in the presence of a catalytical amount of acetic acid (4 mL). The resulting precipitate was filtered off, washed with EtOH and diethyl ether and dried *in vacuum*. The crude product was purified by flash column chromatography (silica gel, CH_2Cl_2:EtOAc, 50/1, v/v) to afford **TTF-TAP-TTF** as a blue solid (16 mg). Yield: 15%. ^{1}H NMR (300 MHz, $CDCl_3$) δ 8.43 (s, 4H), 2.89 – 2.84 (m, 8H), 1.88 (s, 18H), 1.77 – 1.72 (m, 8H), 1.09 – 1.03 (m, 12H). MS (MALDI-TOF, DCTB as matrix, positive) calcd. for $[C_{52}H_{50}N_8S_{12}+H]^+$1171.0880, found 1171.09. HR-MS (ESI, positive): *m/z* calcd for $[C_{52}H_{50}N_8S_{12}+H]^+$: 1171.0880; found: 1171.0872.

Quinoxaline-TAP-TTF. A mixture of 4,5-diaminophthalonitrile (20 mg, 0.13 mmol) and **s-TTF-TAP** (48 mg, 0.06 mmol) in EtOH (15 mL) was refluxed for 20 h in the presence of a catalytical amount of acetic acid (2 mL). The resulting precipitate was filtered off, washed with EtOH and diethyl ether and dried *in vacuum.* The crude product was purified by flash column chromatography (silica gel, CH_2Cl_2:EtOAc,20/1, v/v) to produce **quinoxaline-pyrene-TTF** as a blue solid (19 mg). Yield: 35%. ^{1}H NMR (300 MHz, $CDCl_3$) δ 9.23 (d, J = 5.6 Hz, 2H), 8.26 (s, 2H), 2.89 – 2.84 (m, 4H), 1.89 (d, J = 2.6 Hz, 18H), 1.75 – 1.68(m, 4H) 1.09 – 1.03 (m, 6H). HR-MS (ESI, positive): *m/z* calcd for $C_{44}H_{36}N_{10}S_6$: 896.1443; found: 896.1445.

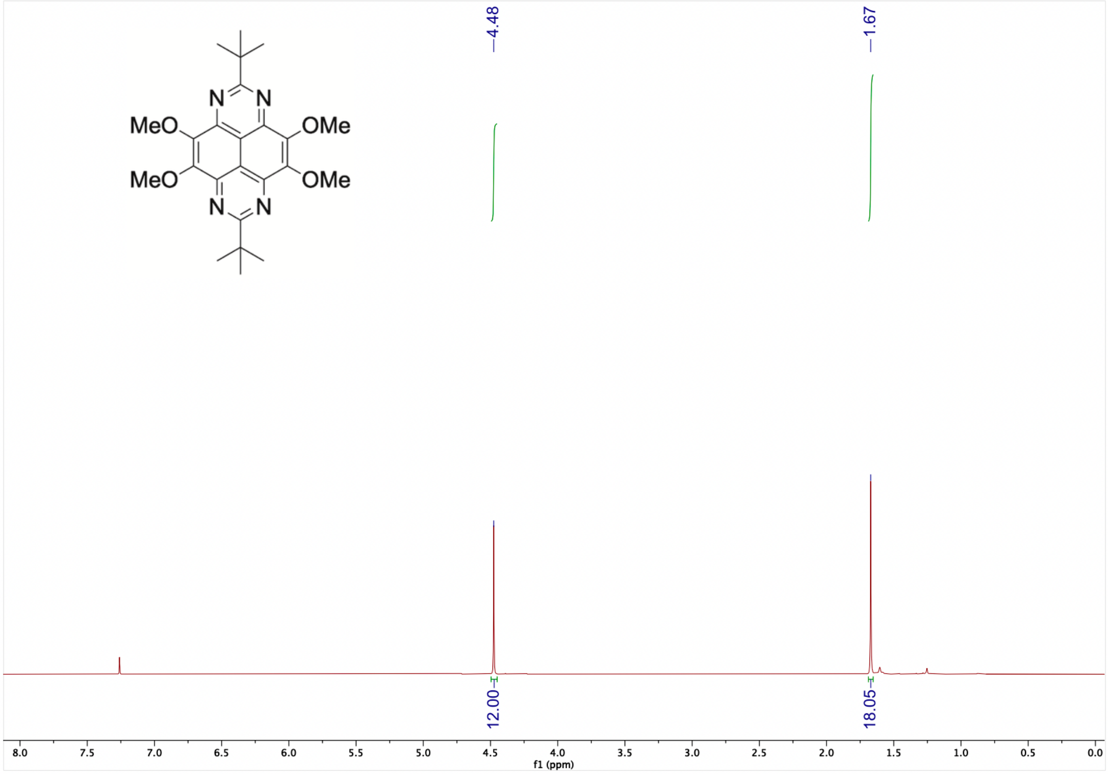

^{1}H NMR spectrum of compound **2** in $CDCl_3$.

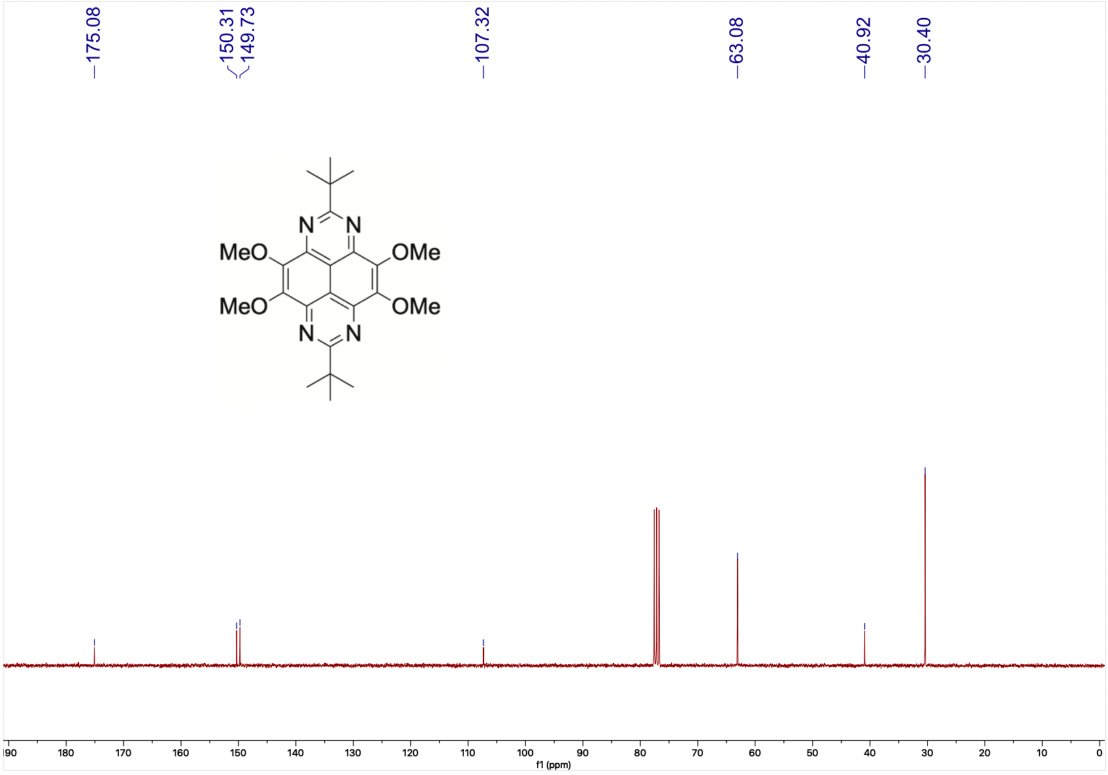

^{13}C NMR spectrum of compound **2** in $CDCl_3$.

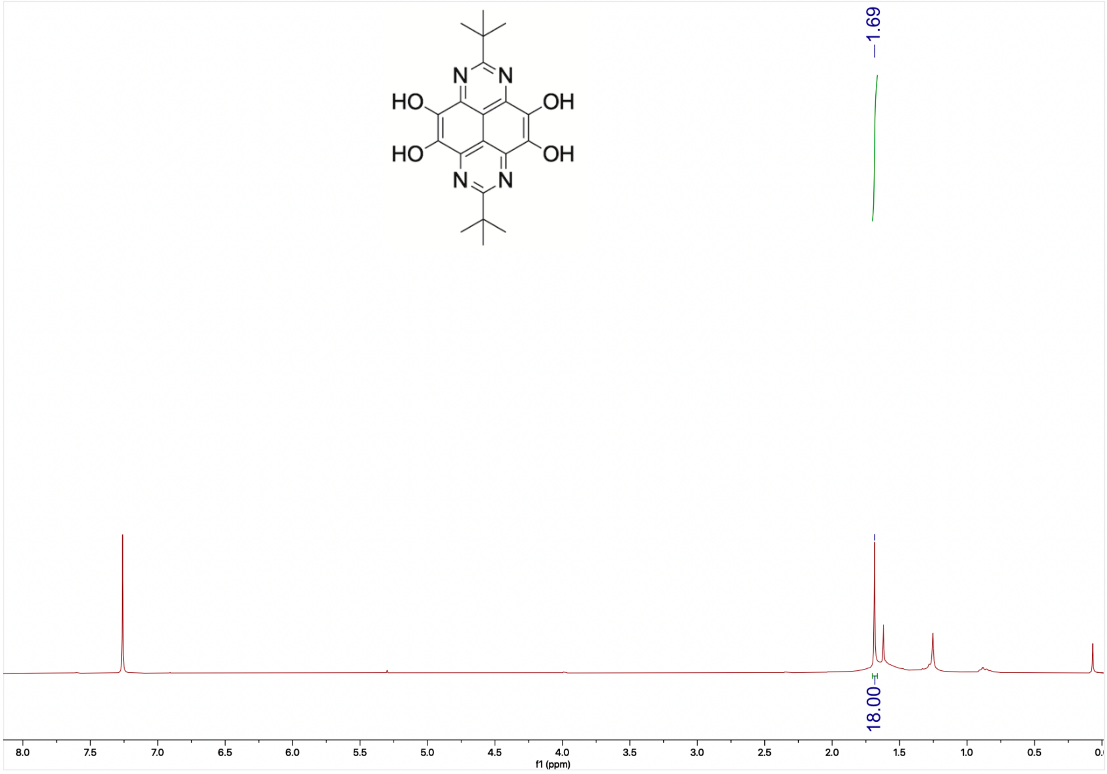

^{1}H NMR spectrum of compound **3** in $CDCl_3$.

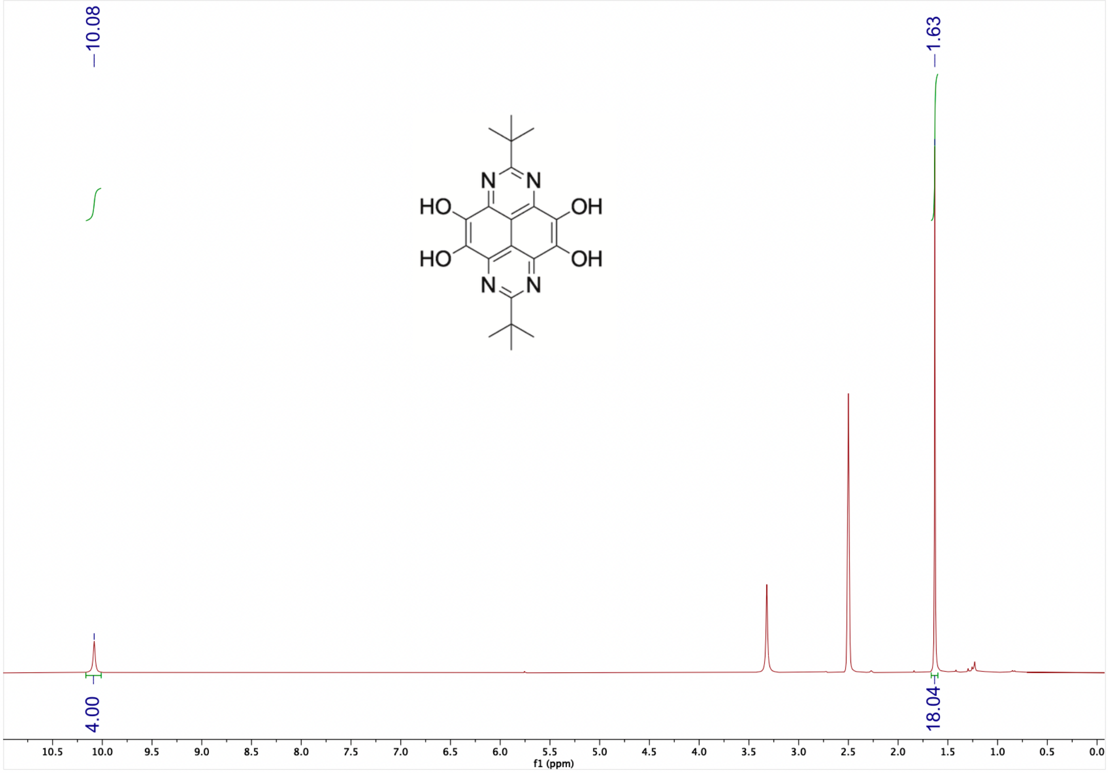

^{1}H NMR spectrum of compound **3** in DMSO-d_6.

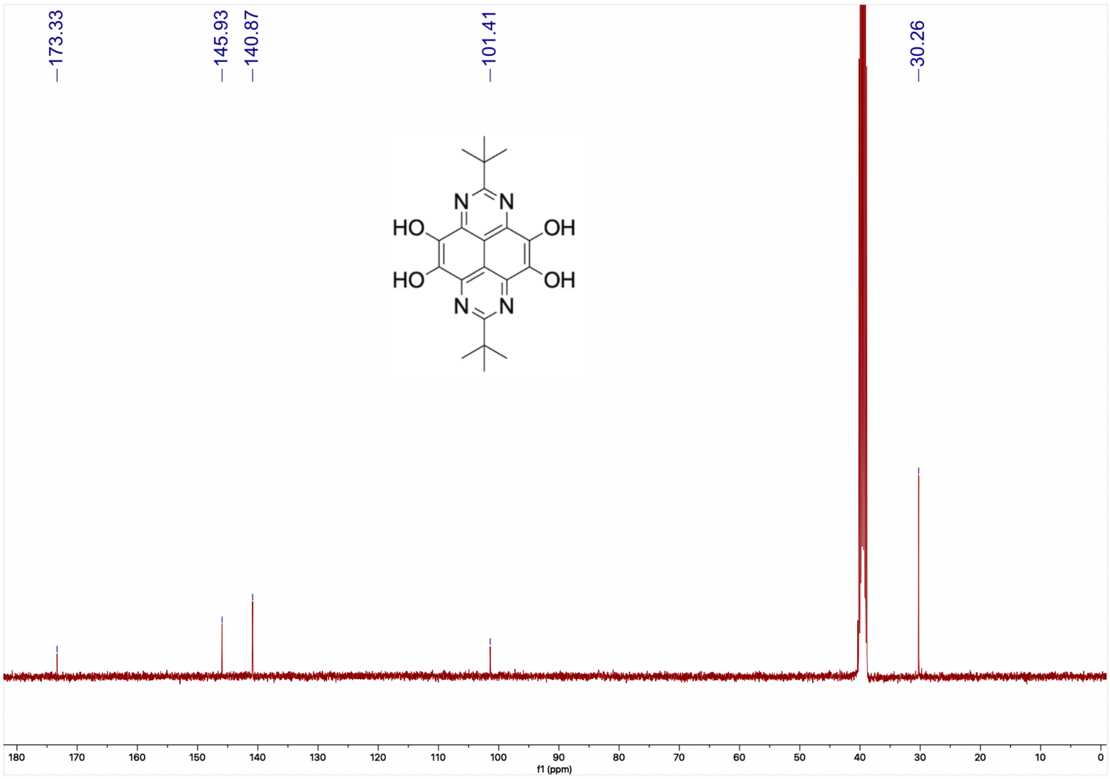

^{13}C NMR spectrum of compound **3** in DMSO-d_6.

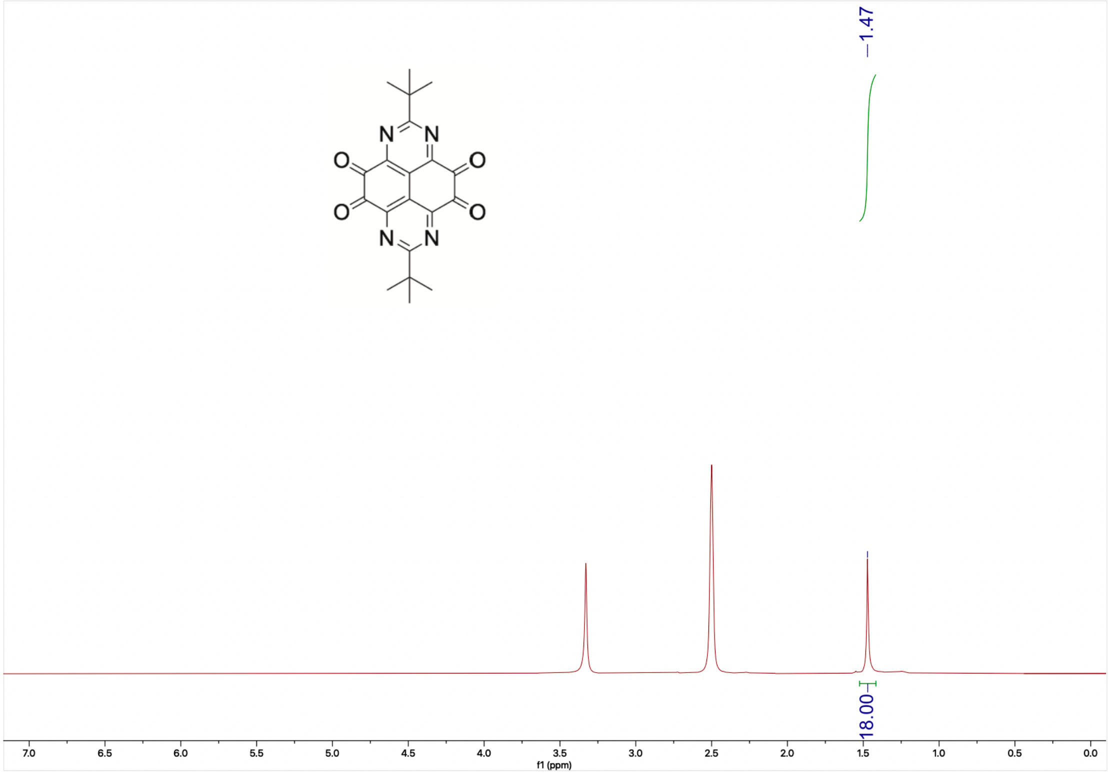

^{1}H NMR spectrum of compound **4** in DMSO-d_6.

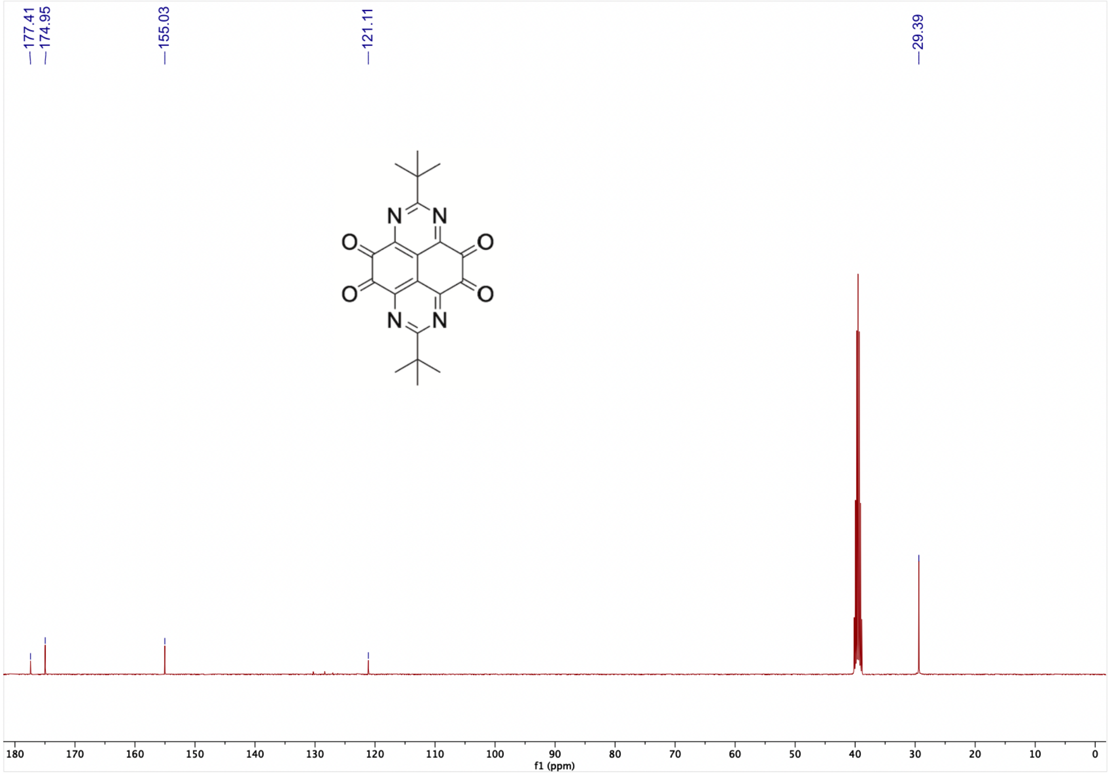

^{13}C NMR spectrum of compound **4** in DMSO-d_6.

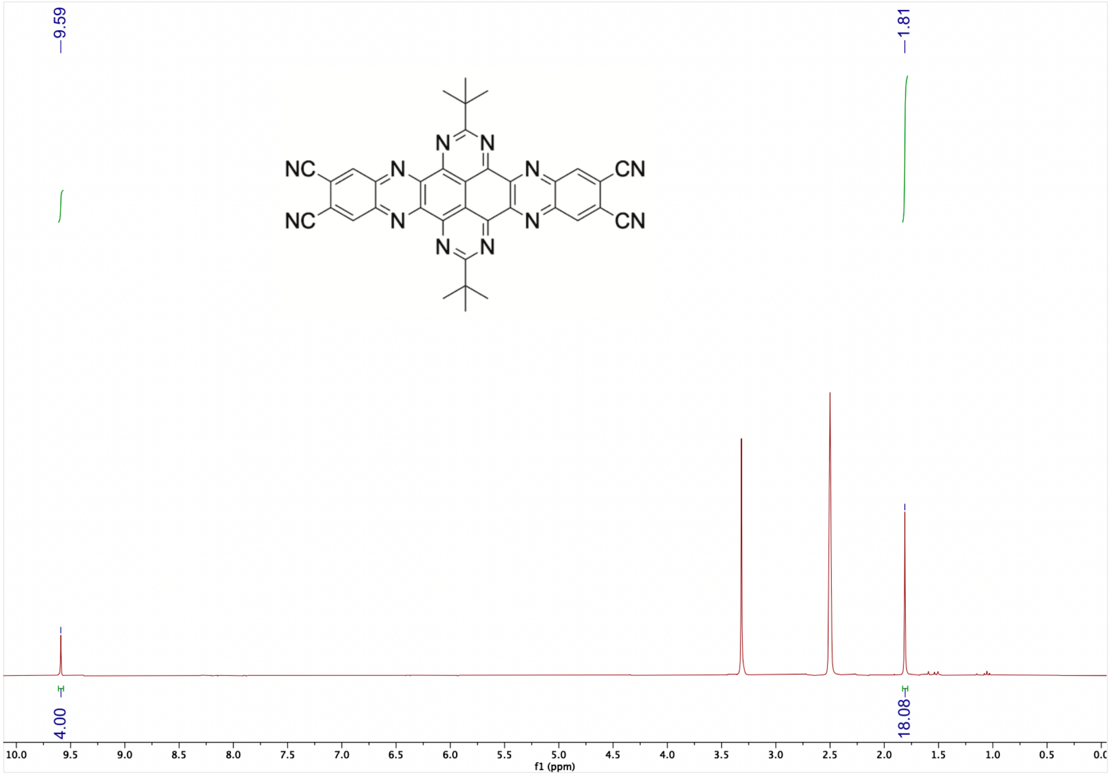

^{1}H NMR spectrum of **diquinoxaline-TAP** in DMSO-d_6.

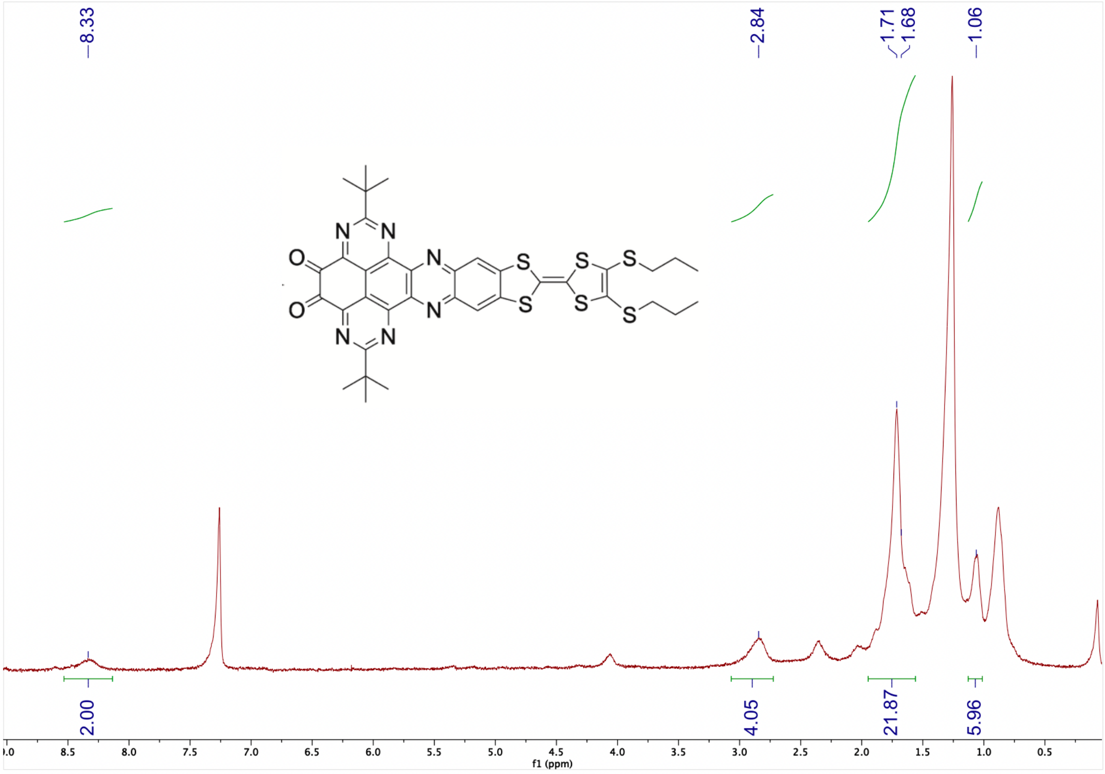

^{1}H NMR spectrum of **s-TTF-TAP** in $CDCl_3$.

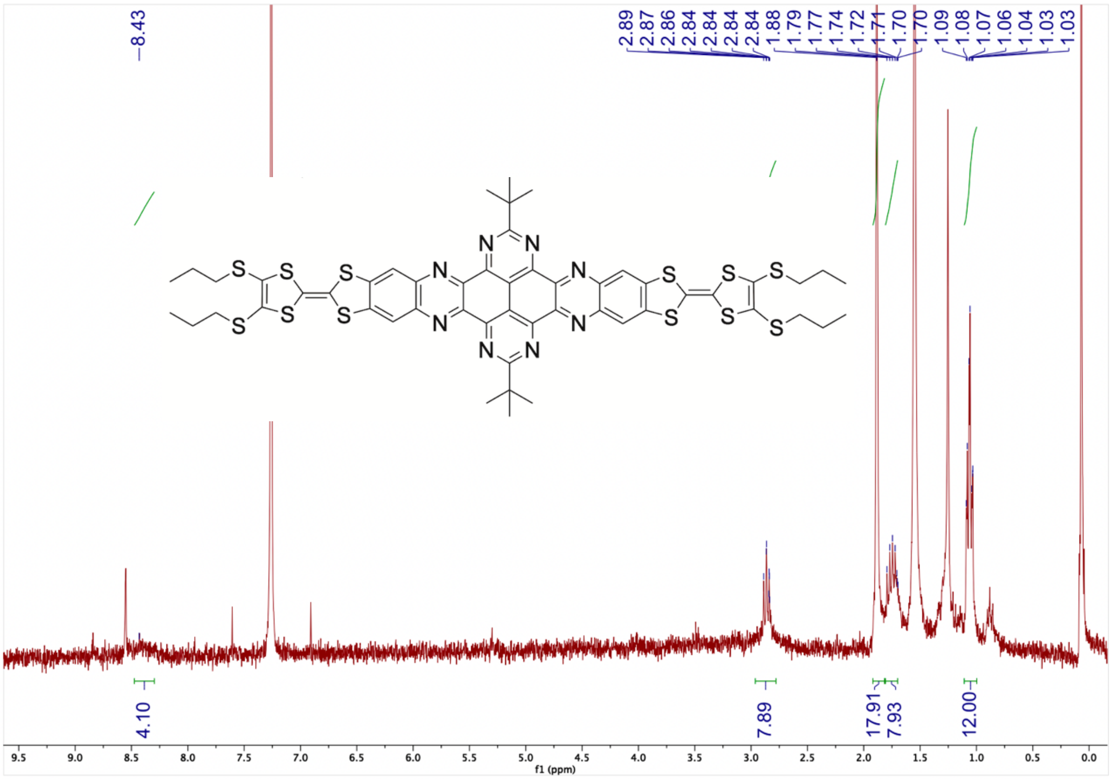

^1H NMR spectrum of **TTF-TAP-TTF** in $CDCl_3$.

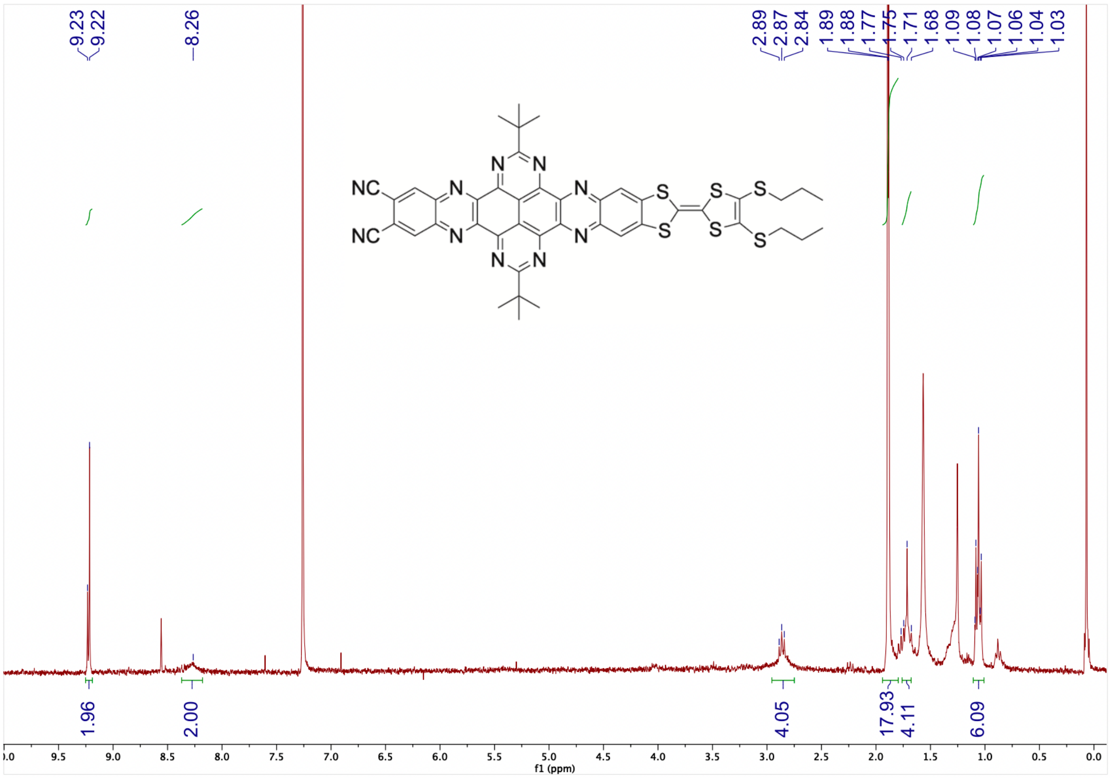

^1H NMR spectrum of **quinoxaline-TAP-TTF** in $CDCl_3$.

Transient absorption measurements. The excitation pulses in the visible region at 580 nm and 605 nm were generated using a tuneable commercial non-collinear optical parametric amplifier (Topas White by Light conversion). UV pulses at 340 nm and 355 nm were produced by second-harmonic generation of visible pulses (680 nm and 710 nm generated by Topas white) with a B-BBO crystal (250 μm thickness). The residual visible radiation was filtered out by a harmonic separator. Both visible and UV excitations pulses were focused to a 60 μm diameter spot ($1/e^2$) and with ~18-20 nJ energy per pulse.

The probe pulse was a broadband continuum ranging from 320 nm to 800 nm and was generated by focusing a fraction of the Ti:Sapphire laser source into a 5 mm thick CaF_2 crystal mounted on a motorized stage, which wobbled horizontally to avoid thermal damage. Then it was focused to a 45 μm diameter spot and overlapped with the pump on the sample. The polarization of the pump with respect to the probe was set at magic angle (54.7°) to measure only population dynamics, excluding rotational ones. A power dependence was regularly carried out to ensure that experiments were conducted in a linear absorption regime. More details on the set up and calculation of the TA signal in a single-shot detection scheme have already been published.[3]

Computational details. To characterize and verify the various electronic transitions, TD-DFT calculations were performed using the Gaussian 16 package[4] at the B3LYP/6-31G(d,p) level of theory, after an initial geometry optimization of all compounds within density functional theory (DFT) using the same functional and basis set. The major transitions of **quinoxaline-TAP-TTF**, **TTF-TAP-TTF** and **diquinoxaline-TAP** are tabulated in Table S1-S3, along with corresponding energy, wavelength, oscillator strength and major molecular orbital contributions.

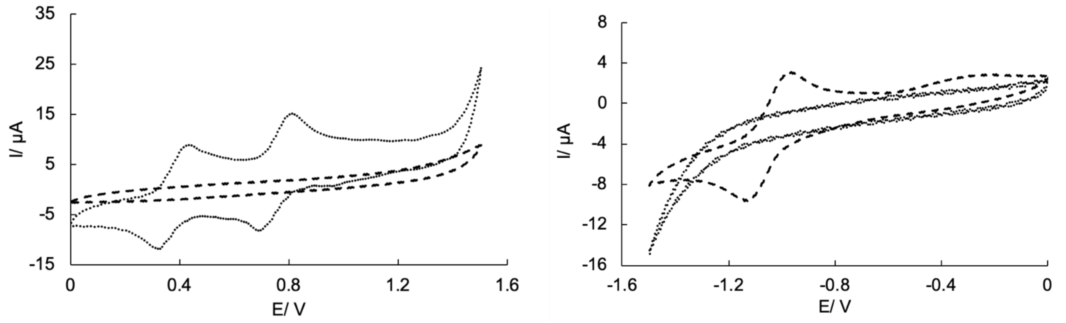

Figure S1. Cyclic voltammograms of **5** (dot) and non-brominated **1** (line) were measured in dichloromethane solution, containing 0.1 M $TBAPF_6$ as the supporting electrolyte at room temperature, a platinum electrode as a working electrode, Ag/AgCl electrode as the reference electrode and the scan rate at 500 mV s^{-1}.

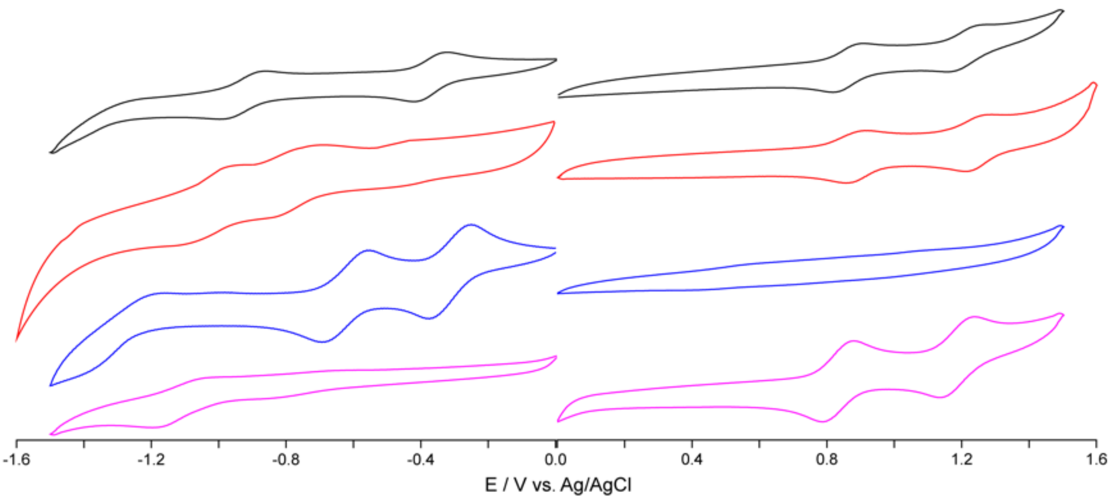

Figure S2. Cyclic voltammograms of **quinoxaline-TAP-TTF** (black), **TTF-TAP-TTF** (red), **diquinoxaline-TAP** (blue) and **s-TTF-TAP** (pink) were measured in dichloromethane solution, containing 0.1 M $TBAPF_6$ as the supporting electrolyte at room temperature, a platinum electrode as a working electrode, Ag/AgCl electrode as the reference electrode and the scan rate at 500 mV s^{-1}.

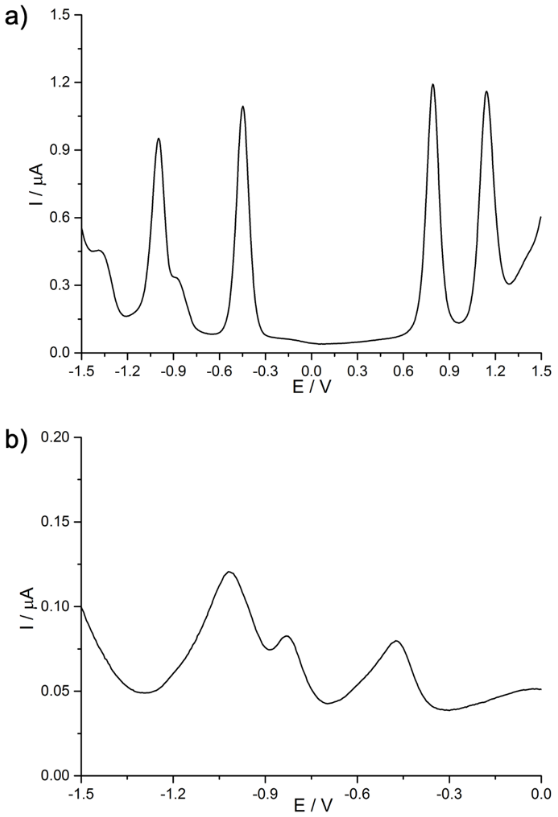

Figure S3. Differential pulse voltammograms of a) **quinoxaline-TAP-TTF** and b) **TTF-TAP-TTF** in DCM at r.t.

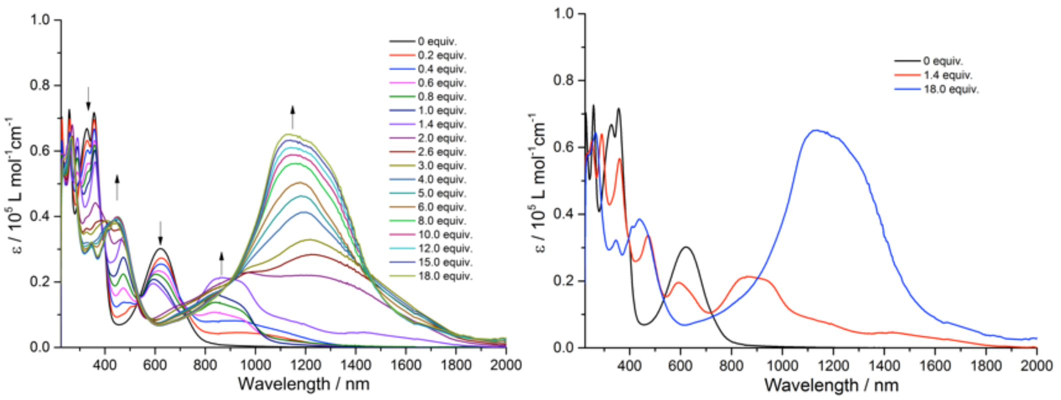

Figure S4. Variation of UV-Vis-NIR absorption spectra of **TTF-TAP-TTF** (5×10^{-6} M) in CH_2Cl_2 upon successive addition of aliquots of $NOSbF_6$ at r.t.

Table S1. Energy, wavelength, oscillator strength, and major molecular orbital contributions to transitions of **quinoxaline-TAP-TTF** according to DFT calculations.

State (S_n)	Energy (cm-1)	λ (nm)	Oscillator strength	Major MO contributions (%)
S_1	12769	783.1	0.0465	HOMO → LUMO (100%)
S_2	16369	610.9	0.0042	HOMO → L+1 (99%)
S_3	16549	604.3	0.3249	HOMO → L+2 (99%)
S_{13}	25556	391.3	0.0148	H-6 → LUMO (43%), H-1 → L+2 (45%)
S_{14}	25707	389.0	0.2463	H-1 → L+1 (79%)
S_{20}	27280	366.6	0.0275	HOMO → L+6 (96%)
S_{21}	27777	360.0	0.1264	H-2 → L+2 (19%), HOMO → L+4 (68%)
S_{22}	27894	358.5	0.2457	H-2 → L+1 (21%), H-2 → L+2 (38%) HOMO → L+4 (23%)
S_{23}	27902	358.4	0.1236	H-7 → L+1 (10%), H-2 → L+1 (49%) H-2 → L+2 (19%)
S_{25}	28593	349.7	0.1337	H-10 → LUMO (35%), H-6 → L+1 (26%)
S_{27}	28826	346.9	0.2416	H-3 → L+2 (79%)
S_{28}	28937	345.6	0.0905	H-3 → L+1 (76%)
S_{31}	29712	336.6	0.023	H-6 → L+2 (86%)
S_{33}	30701	325.7	0.5147	H-6 → L+1 (16%), H-5 → L+2 (51%)
S_{34}	31032	322.2	0.0183	H-6 → L+1 (21%), H-5 → L+2 (30%) HOMO → L+7 (16%)
S_{35}	31109	321.5	0.0173	H-5 → L+1 (47%), HOMO → L+8 (35%)
S_{36}	31151	321.0	0.0967	H-6 → L+1 (17%), HOMO → L+7 (63%)
S_{38}	32545	307.3	0.1045	H-13 → LUMO (62%), H-12 → LUMO (16%)
S_{40}	33094	302. 2	0.4082	H-1 → L+3 (13%), HOMO → L+9 (14%) HOMO → L+10 (27%), HOMO → L+12 (23%)

Table S2. Energy, wavelength, oscillator strength (> 0.01), and major molecular orbital contributions to transitions of **TTF-TAP-TTF** according to DFT calculations.

State (S_n)	Energy (cm-1)	λ (nm)	Oscillator strength	Major MO contributions (%)
S_1	16447	608.0	0.6215	H-1 → LUMO (15%), HOMO → LUMO (79%)
S_3	18842	530.7	0.131	H-1 → L+1 (37%), HOMO → L+1 (56%)
S_4	18941	528.0	0.0209	H-1 → L+1 (57%), HOMO → L+1 (39%)
S_6	19511	512.5	0.0124	H-1 → L+2 (86%), HOMO → L+2 (13%)
S_{16}	26775	373.5	0.135	H-6 → LUMO (13%), H-2 → L+2 (67%)
S_{17}	26897	371.8	0.0319	HOMO → L+5 (37%), HOMO → L+6 (46%)
S_{18}	26920	371.5	0.0292	H-1 → L+5 (49%), H-1 → L+6 (35%)
S_{19}	27173	368.0	0.0199	H-3 → LUMO (35%), H-2 → L+1 (56%)
S_{20}	27328	365.9	0.0025	H-5 → LUMO (60%), H-3 → L+2 (19%)
S_{21}	28182	354.8	1.2807	H-6 → LUMO (64%), H-2 → L+2 (20%)
S_{22}	28217	354.4	0.026	H-13 → LUMO (29%), H-3 → L+1 (60%)
S_{28}	29261	341.8	0.1434	H-8 → LUMO (79%)
S_{32}	29844	335.1	0.6648	H-5 → L+1 (77%)
S_{34}	29913	334.3	0.0107	HOMO → L+3 (57%), HOMO → L+7 (34%)
S_{37}	30081	332.4	0.0201	H-1 → L+4 (25%), H-1 → L+7 (29%), HOMO → L+3 (20%), HOMO → L+7 (21%)

Table S3. Energy, wavelength, oscillator strength, and major molecular orbital contributions to transitions of **diquinoxaline-TAP** according to DFT calculations.

State (S_n)	Energy (cm-1)	λ (nm)	Oscillator strength	Major MO contributions (%)
S_7	25932	385.6	0.0358	H-1 → L+1 (95%)
S_9	27101	369.0	0.0773	H-4 → L+1 (12%), H-1 → L+2 (76%)
S_{19}	30110	332.1	1.4482	H-7 → LUMO (60%), H-1 → L+2 (17%)
S_{20}	30593	326.9	0.2322	H-7 → LUMO (21%), H-4 → L+1 (73%)
S_{23}	32658	306.2	0.013	H-8 → L+1 (89%)
S_{26}	33202	301.2	0.1551	H-4 → L+2 (83%)
S_{30}	34485	290.0	0.8118	H-8 → L+2 (86%)
S_{39}	37114	269.4	0.0266	H-11 → LUMO (93%)

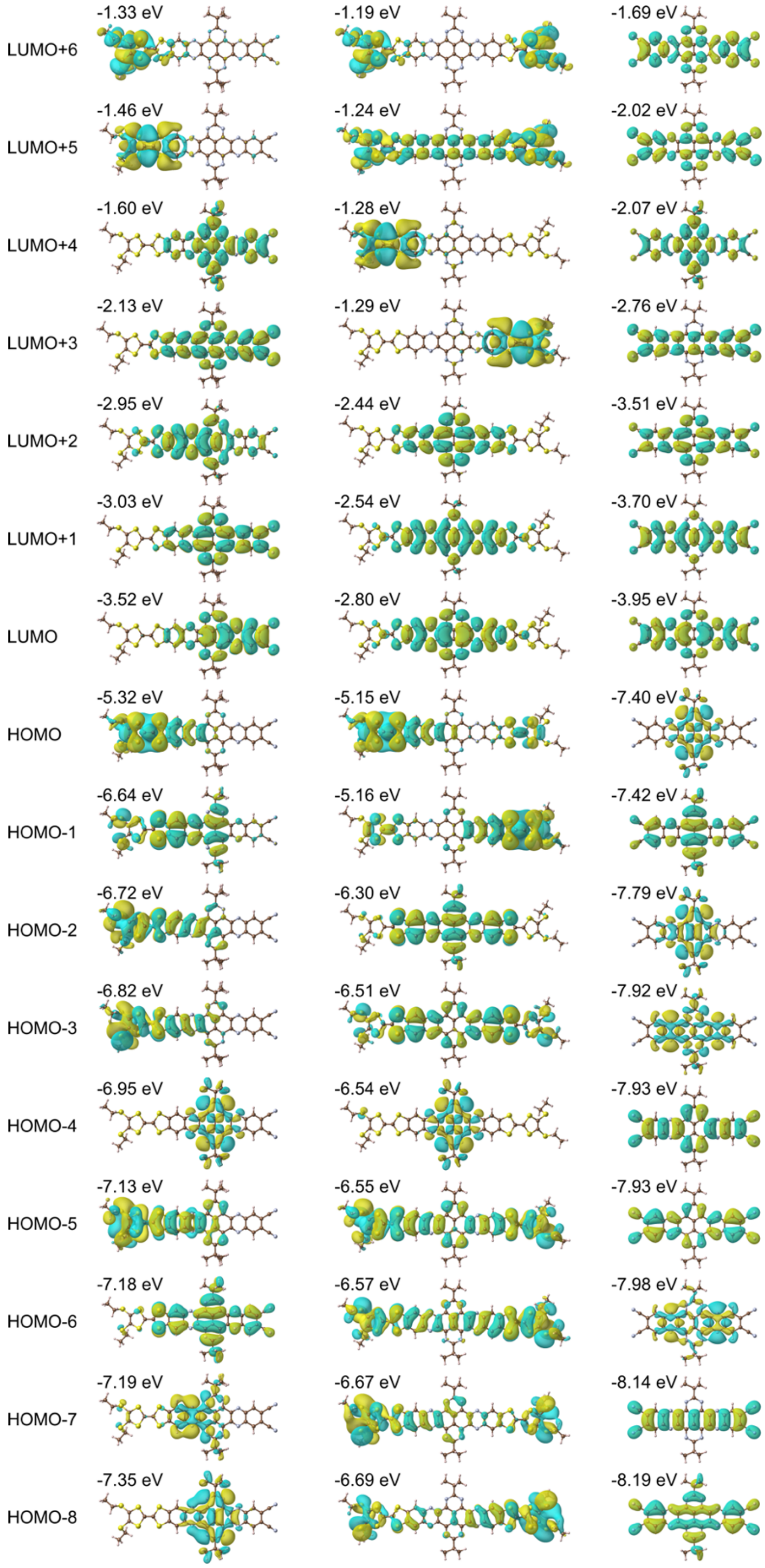

Figure S5. The molecular orbitals involved in the main transitions of **quinoxaline-TAP-TTF** (left), **TTF-TAP-TTF** (middle) and **diquinoxaline-TAP** (right), respectively.

Electrostatic potential of the S_1 state for different molecules and conformations

As shown in Figure S6, in the initial excited S_1 state of TTF-TAP-TTF an electron is somewhat more favorably located on the left TTF (around 7Å) than the right one (around 24Å) but the electrostatically most favorable location is on the TAP core (around 20Å). This contrasts with **quinoxaline-TAP-TTF** as well as another asymmetric test molecule **OMe-TAP-TTF**, where an electron is more favorably located on the left TTF (thus recombining with the hole) than on the TAP core. For **TTF-TAP-TTF** the left TTF and thus recombination only becomes favorable upon structural relaxation of the S_1 state. This can be explained with a partial hole localization on both TTF units in **TTF-TAP-TTF** in the S_1 state that electrostatically stabilizes the excited electron in the center, i.e. on the TAP core. Upon structural relaxation of the S_1 state, the hole localizes more on the left TTF and this stabilization is reduced.

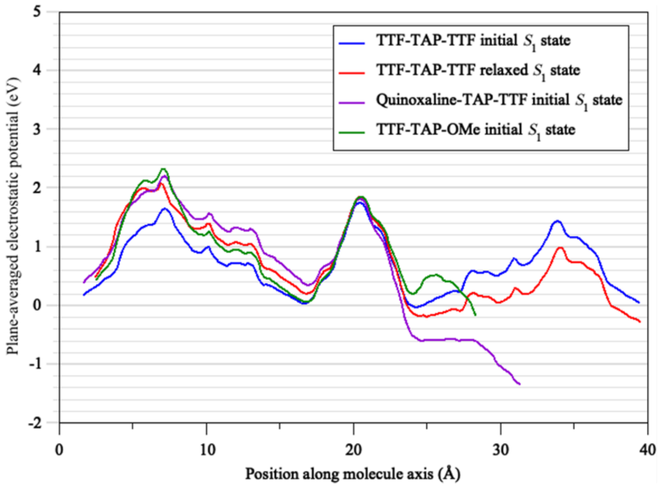

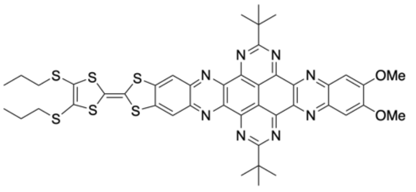

TTF-TAP-OMe

Figure S6. (left) Plane averaged-electrostatic potential in the excited state along the long molecular axis. Higher potential values imply more favourable locations for a probe electron. (right) chemical structure of TTF-TAP-OMe.

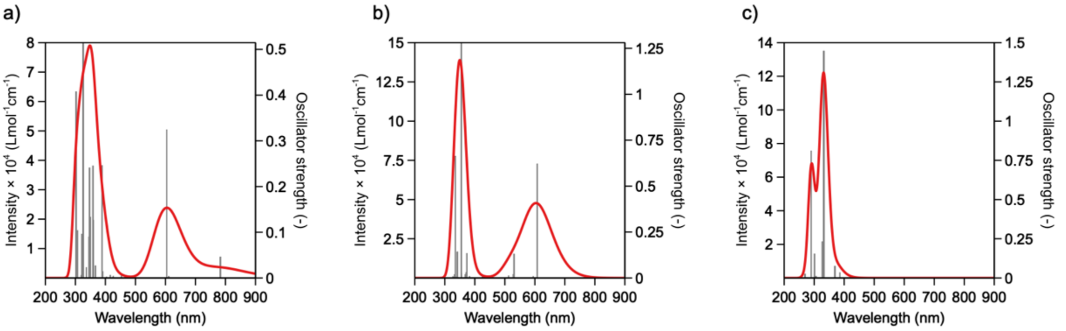

Figure S7. Computed absorption spectra and oscillator strengths of a) **quinoxaline-TAP-TTF**, b) **TTF-TAP-TTF** and c) **diquinoxaline-TAP**.

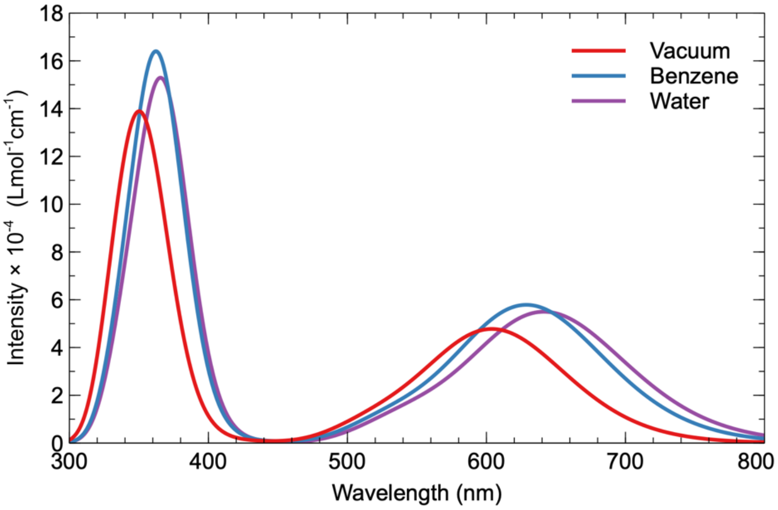

Figure S8. Computed absorption spectra of **TTF-TAP-TTF** in vacuum and different solvents.

It is worth noting that data in the main text was computed in vacuum and that in general a slight red shift is observed in solvents, slightly more so in polar solvents as shown in Fig. S8.

Data analysis of time-resolved absorption spectra.

We used the so-called Singular Value Decomposition and Global Fit (SVD-GF) analysis of the transient absorption data, $TA(\lambda, t)$, to separate noise, $\Xi(\lambda, \mathrm{t})$, from the spectral evolution of the system, $\widehat{TA}(\lambda, t)$.

$$\mathrm{TA}(\lambda, t) = \widehat{TA}(\lambda, t) + \Xi(\lambda, t) \qquad \text{eq. 1}$$

Assuming that the dynamics can be described by exponential decays, the SVD-GF analysis allows to decompose $\widehat{TA}(\lambda, t)$ as a series of exponential decays with characteristic lifetimes (τ_k) and decay associated spectra, DASs:

$$\widehat{TA}(\lambda, t) = \sum_{k=1}^{k=p} DAS_k(\lambda)\, e_{t>0}^{-\frac{t}{t_k}} |_{IRF(K,t_0,t)} \qquad \text{eq. 2}$$

Where $e_{t>0}^{-t/\tau_k} |_{IRF(K,t_0,t)}$ represents an exponential decay with decay constant τ_k multiplied by the Heaviside step function $u_0(t)$ and convoluted with the instrument response function (IRF). The latter is assumed to be a Gaussian centred at t_0 and with a full width at half maximum of *K*.

Ultrafast investigation of the diquinoxaline-TAP upon UV (355 nm) excitation

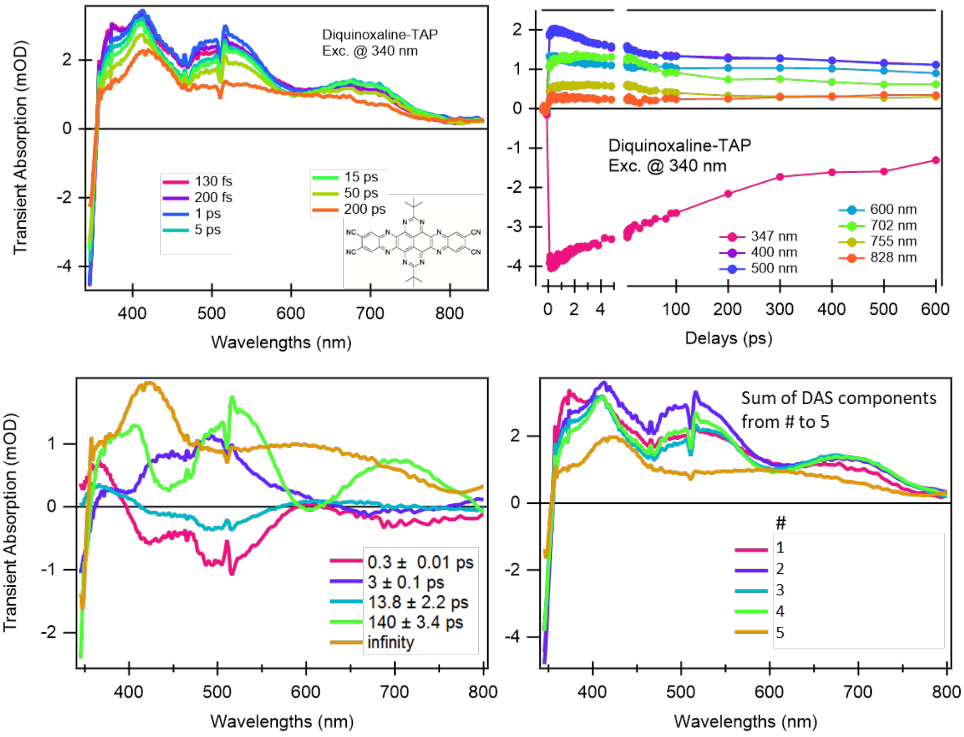

Figure S9. Ultrafast investigation of the **diquinoxaline-TAP** upon UV (355 nm) excitation. (top, left) Selected transient absorption spectra; (Top right) Kinetic traces at representative probe wavelengths. (Bottom) outcome of the SVD-GF analysis: (left) decay associated spectra (DAS) and (right) their sum.

Figure S9 summarises the investigation of the **diquinoxaline-TAP** upon excitation UV at 355 nm (no absorption in the visible is present). A detailed discussion of the photophysics of this system is not the main topic of this work but we can shortly describe the outcomes. First, the system undergoes several spectral changes that are not compatible with mere cooling and solvation dynamics: more specifically the first spectrum shows a peak at 375 that decays within 1 ps, which is accompanied by a rise of the ESA band at 500 nm (see relative kinetics in the top right panel). This result, which is fully confirmed by the SVD-GF analysis (see red curves in the bottom panels), is more compatible with an electronic relaxation rather than a conformational change because of the inherent rigidity of the system. The following relaxation is mainly a decay of the 500 nm ESA band in 3 ps. The 13 ps component is probably a deviation of the 3 ps decay from a perfect exponential behavior. The behavior in the hundreds of ps range is rather complex since we observed three distinctive ESA bands at 400 nm, 520 nm and 700 nm, which all decay in 140 ps. Despite it is very likely also present in this time range, this behavior is not compatible with mere rotational diffusion dynamics. More important, after these decays, the spectral shape is completely different indicating that the long-lived state is electronically different from the initially excited ones. All these dynamics, except the 0.3 ps one, are accompanied by a GS recovery.
This qualitative analysis points to a photocycle more complex than observed for typical molecules. This could be in agreement with a recent publication,[5] where we observed in quinoxdt Pt dithiolene complexes that the lifetime of the S_2 state, which is dominantly due to the LUMO+1 localized on the quinoxaline ligand, can be as long as almost 2 ps (but typically few hundreds of fs). Accordingly, we could assign the 0.3 ps to the $S_2 \rightarrow S_1$ internal conversion; the 3 ps and 13 ps component to conformational dynamics in S_1 state, and the 140 ps ISC toward a low-lying triplet state.
More relevant to the article, since it has no absorption in the visible and its ESA signal at 550 -650 nm is rather flat, the observed GSB dynamics, as discussed in the main text, can safely be ascribed to the ICT dynamics. The negative peak at 375 nm observed in the TTF containing molecules is absent, confirming that it is due to oxidized TTF. The ESA band at 500 nm overlaps with the ESA bands from oxidized TTF, but the dynamics are so different that we can exclude the presence of any signal due to excited TAP. In conclusion, we can safely state that the spectra reported in the main text are due to photo-excited ICT states and photo-oxidized TTF, without contamination due to excited TAP moieties also upon UV excitation.

Analysis of the transient absorption measurements of the quinoxaline-TAP-TTF upon visible (580 nm) excitation

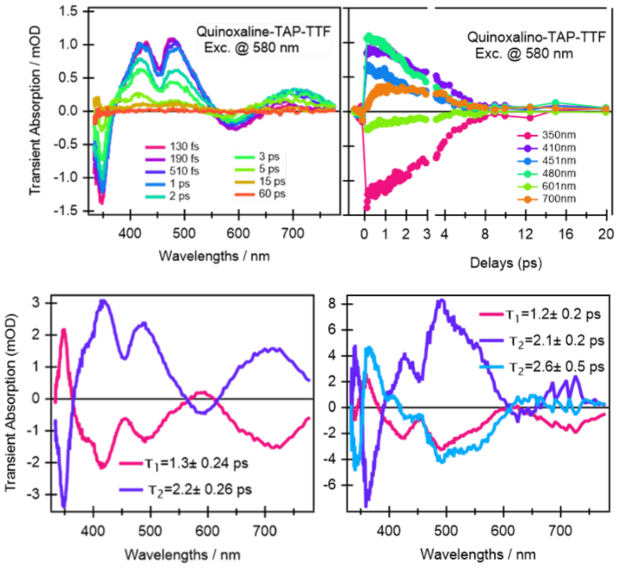

Figure S10. Ultrafast investigation of the **quinoxaline-TAP-TTF** upon 580 nm excitation. (top raw, from Figure 4) selected transient absorption spectra (left) and kinetic traces (right) at representative probe wavelengths. (Bottom) outcome of the SVD-GF analysis: decay associated spectra (DAS) assuming two (left) and three (right) components.

Figure S10 summarises the investigation of the **quinoxaline-TAP-TTF** upon excitation at 580 nm. The analysis reported in the bottom line, reveals that, regardless the number of exponential components, we always end up in spectra that look inversed of each other and with close time constants. At first superficial glance, it could be rationalized by assuming a rise of the overall signal due to the formation of the ICT state, followed by a charge recombination in 2-3 ps, but this is in contradiction with the direct nature of the ICT transition. Instead, as aforesaid, this is due to the limitation of the SVD GF analysis in describing non-exponential dynamics or exponential decays with close time constants. Indeed, as commented before, the signal decays non-exponentially at any wavelength. This agrees with the occurrence of several processes with opposite effects on the signal strength, such as solvation, cooling, conformational relaxation, stabilization of the charge separation state, etc., on the same timescale as the back charge transfer. This is also in agreement with the analysis of the **TTF-TAP-TTF** (Figure 5), where the 700 fs DAS was assigned to the solvation and stabilization of the ICT state.

Accordingly, the spectral changes described by the 1.3 ps DAS (absorption at 350 and 600 nm and negative signals at 420, 500 and 700 nm) are due to a modulation of the CT absorption decay, described by the 2.2 ps component, by these other relaxation processes occurring on the same time scale. The effect is the non-exponential (almost linear) decay reported in Figure S11. Such a condition, with non-exponential decays and a modulation of the back CT process on comparable time scales, is where a global-fitting with exponential decays shows its limitation. In this sense we do not consider the 1.3 ps component as an artefact but the way how such complex dynamics can be

described within the assumption of exponentially decaying components. In particular the value of 1.3 ps has the meaning of an average time scale of the processes competing with the back CT one, in agreement with the presence of the 700 fs DAS (Figure 5) in **TTF-TAP-TTF.**

It is worth mentioned that we also consider the possibility to use a non-exponential decay basis set (as for instance stretched exponentials or assuming a non-Markovian behaviour, where the rate constants are time dependent) but we considered such approach rather arbitrary and of difficult rationalization and modelling. For this reason, we decided to keep the more conventional analysis, which assume a linear combination of exponential decays, and making the reader aware that the 1.3ps DAS describe a modulation of the second DAS rather than a real associated spectrum.

Selected kinetic traces from quinoxaline-TAP-TTF and from TTF-TAP-TTF upon visible and UV excitation

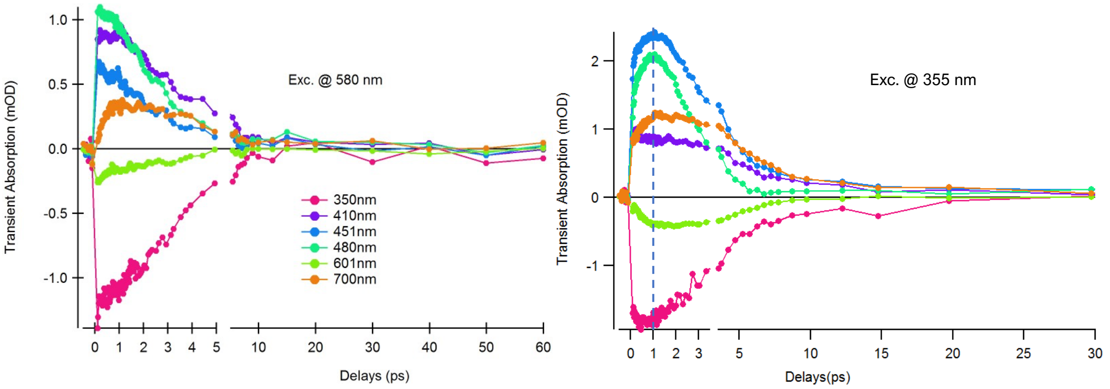

Figure S11. Selected kinetic traces from **quinoxaline-TAP-TTF** upon excitation at 580 nm (from Figure 4) and 355 nm, respectively. The comparison shows a rise of the signals in the UV excited sample occurring in the first 1 ps.

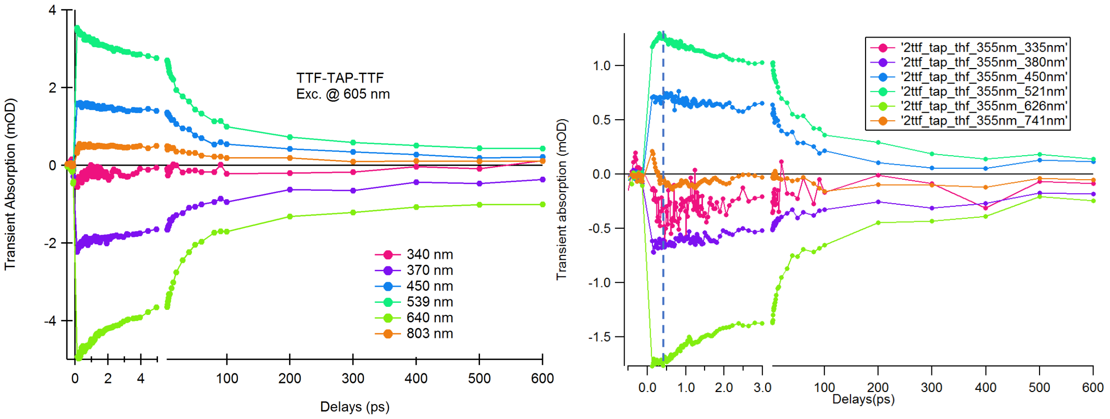

Figure S12. Selected kinetic traces from **TTF-TAP-TTF** upon excitation at 605 nm and 355 nm, respectively. The comparison shows a rise of the signals in the UV excited sample occurring in the first 0.5 ps.

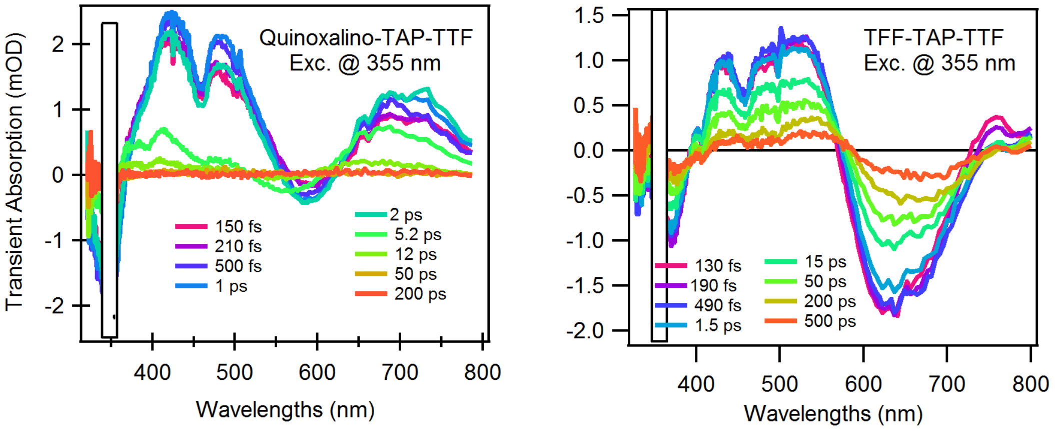

Figure S13. Selection of ultrafast transient absorption spectra of **quinoxaline-TAP-TTF** and **TTF-TAP-TTF** upon excitation at 355 nm.

For the sake of completeness and to answer the relevant question whether the ICT state could also be populated indirectly upon excitation at shorter wavelength, we carried out the same experiment upon excitation of TTF- and TAP-centered states at 355 nm. We find the same spectral signatures as shown in Figure S13 but with a significant difference in the kinetic evolution (Figure S11 and Figure S12). In **quinoxaline-TAP-TTF** we observe a rise of the ICT GSB at 600 nm along with TTF oxidation signal at 410 nm and 480 nm (see respective kinetic traces in Figure S11). The rise of ICT GSB indicates an indirect population of TTF→TAP transition in 200 to 250 fs. Moving the **TTF-TAP-TTF**, oxidized TTF signal (ESA bands at 530-480 nm) shows a similar rise as **quinoxaline-TAP-TTF** but faster. No further change in the following dynamics in both samples can be observed in comparison with measurements directly triggering ICT transition. Small differences in dynamics of **quinoxaline-TAP-TTF** observed at 500 nm and 700 nm upon excitation at 355 and 580 nm are due to TAP contribution in a time scale of 500 fs, (see the 300 fs DAS in Figure S13, which shows indeed two prominent bands at 500 nm and 700 nm).

In short, the ICT state can also be indirectly populated upon excitation of TTF- and TAP-localized states at 355 nm for 130 fs and 250 fs in **TTF-TAP-TTF** and **quinoxaline-TAP-TTF**, respectively, since the spectral shapes in both cases resemble more the late ones upon the direct excitation to ICT states.

From the comparison of the absorption spectra in Fig. 1, it appears that the spectral region around 355 nm should be from TAP-centered transitions, namely on the acceptor, with a minor contribution from the TTF-centered transitions. Accordingly, the 130 and 250 fs components could be rationalized as a hole transfer to the HOMO of the donor following the excitation of the acceptor.

Species Associated Spectra (SASs) and Evolution-Associated Differential Spectra (EADS)[6] for the TTF-TAP-TTF

Figure 6 in the main text shows the proposed model of the relaxation processes for the **TTF-TAP-TTF**. In this scheme the relaxation processes follow a sequential path from the initially exited state to the C state. However, because of the presence of branching to the GS from any state, this is not a pure sequential model. For the sake of completeness, we report in Figure S14 the corresponding Species Associated Spectra and the Evolution-Associated Differential Spectra (EADS) where the relaxation model is purely sequential in the absence of branching.

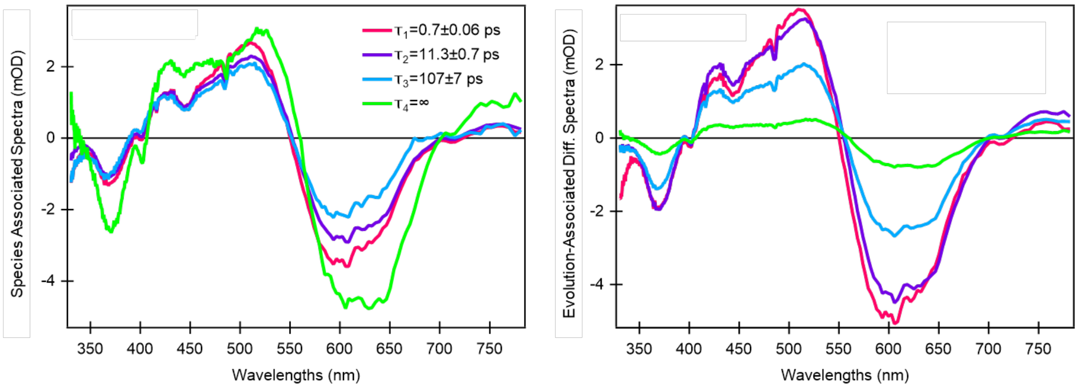

Figure S14. Relaxation model from figure 6 used for the calculation of the SAS (left panel) and the EADS (right panel) for **TTF-TAP-TTF**

References

1. P. Zhou, U. Aschauer, S. Decurtins, T. Feurer, R. Häner and S. X. Liu, *Chem. Commun.*, 2021, **57**, 12972.
2. C. Jia, S.-X. Liu, C. Tanner, C. Leiggener, A. Neels, L. Sanguinet, E. Levillain, S. Leutwyler, A. Hauser and S. Decurtins, *Chem. Eur. J.*, 2007, **13**, 3804.
3. M. Nazari Haghighi Pashaki, N. Mosimann-Schönbächler, A. Riede, M. Gazzetto, A. Rondi and A. Cannizzo, *J. Phys. Photonics*, 2021, **3**, 034014.
4. Gaussian 16 Rev. C.01, M. J. Frisch, G. W. Trucks, H. B. Schlegel, G. E. Scuseria, M. A. Robb, J. R. Cheeseman, G. Scalmani, V. Barone, G. A. Petersson, H. Nakatsuji, X. Li, M. Caricato, A. V. Marenich, J. Bloino, B. G. Janesko, R. Gomperts, B. Mennucci, H. P. Hratchian, J. V. Ortiz, A. F. Izmaylov, J. L. Sonnenberg, Williams, F. Ding, F. Lipparini, F. Egidi, J. Goings, B. Peng, A. Petrone, T. Henderson, D. Ranasinghe, V. G. Zakrzewski, J. Gao, N. Rega, G. Zheng, W. Liang, M. Hada, M. Ehara, K. Toyota, R. Fukuda, J. Hasegawa, M. Ishida, T. Nakajima, Y. Honda, O. Kitao, H. Nakai, T. Vreven, K. Throssell, J. A. Montgomery Jr., J. E. Peralta, F. Ogliaro, M. J. Bearpark, J. J. Heyd, E. N. Brothers, K. N. Kudin, V. N. Staroverov, T. A. Keith, R. Kobayashi, J. Normand, K. Raghavachari, A. P. Rendell, J. C. Burant, S. S. Iyengar, J. Tomasi, M. Cossi, J. M. Millam, M. Klene, C. Adamo, R. Cammi, J. W. Ochterski, R. L. Martin, K. Morokuma, O. Farkas, J. B. Foresman and D. J. Fox, Wallingford, CT, 2016.

5. M. Gazzetto, F. Artizzu, S. S. Attar, L. Marchiò, L. Pilia, E. J. Rohwer, T. Feurer, P. Deplano and A. Cannizzo, *J. Phys. Chem. A*, 2020, **124**, 10687.
6. I. H. M. van Stokkum, D. S. Larsen and R. van Grondelle, *Biochim. Biophys. Acta - Bioenerg.*, 2004, **1657**, 82.
